# The CodY-dependent *clhAB2* operon is involved in cell shape, chaining and autolysis in *Bacillus cereus* ATCC 14579

**DOI:** 10.1371/journal.pone.0184975

**Published:** 2017-10-09

**Authors:** Eugénie Huillet, Ludovic Bridoux, Pagakrong Wanapaisan, Agnès Rejasse, Qi Peng, Watanalai Panbangred, Didier Lereclus

**Affiliations:** 1 Micalis Institute, INRA, AgroParisTech, Université Paris-Saclay, Jouy-en-Josas, France; 2 Department of Biotechnology, Faculty of Science, Mahidol University, Bangkok, Thailand; Institut Pasteur, FRANCE

## Abstract

The Gram-positive pathogen *Bacillus cereus* is able to grow in chains of rod-shaped cells, but the regulation of chaining remains largely unknown. Here, we observe that glucose-grown cells of *B*. *cereus* ATCC 14579 form longer chains than those grown in the absence of glucose during the late exponential and transition growth phases, and identify that the *clhAB*_*2*_ operon is required for this chain lengthening phenotype. The *clhAB*_*2*_ operon is specific to the *B*. *cereus* group (i.e., *B*. *thuringiensis*, *B*. *anthracis* and *B*. *cereus*) and encodes two membrane proteins of unknown function, which are homologous to the *Staphylococcus aureus* CidA and CidB proteins involved in cell death control within glucose-grown cells. A deletion mutant (Δ*clhAB*_*2*_) was constructed and our quantitative image analyses show that Δ*clhAB*_*2*_ cells formed abnormal short chains regardless of the presence of glucose. We also found that glucose-grown cells of Δ*clhAB*_*2*_ were significantly wider than wild-type cells (1.47 μm ±CI_95%_ 0.04 vs 1.19 μm ±CI_95%_ 0.03, respectively), suggesting an alteration of the bacterial cell wall. Remarkably, Δ*clhAB*_*2*_ cells showed accelerated autolysis under autolysis-inducing conditions, compared to wild-type cells. Overall, our data suggest that the *B*. *cereus clhAB*_*2*_ operon modulates peptidoglycan hydrolase activity, which is required for proper cell shape and chain length during cell growth, and down-regulates autolysin activity. Lastly, we studied the transcription of *clhAB*_*2*_ using a *lacZ* transcriptional reporter in wild-type, *ccpA* and *codY* deletion-mutant strains. We found that the global transcriptional regulatory protein CodY is required for the basal level of *clhAB*_*2*_ expression under all conditions tested, including the transition growth phase while CcpA, the major global carbon regulator, is needed for the high-level expression of *clhAB*_*2*_ in glucose-grown cells.

## Introduction

In *Staphylococcus aureus*, the CidR regulon is necessary for optimal survival in late stationary phase cultures and during biofilm development in the presence of excess glucose (~0.6%) [1–3]. Under these growth conditions, the CidR regulon is involved in the down-regulation of acetate production, which reduces cytoplasmic acidification and ultimately limits cell death and lysis [1,2]. The CidR transcriptional regulator activates the expression of two operons including *cidABC* and *alsSD* that display pro- and anti-death functions, respectively [3]. The *cidABC* operon encodes the CidA and CidB integral membrane proteins and the CidC pyruvate oxidase and the expression of *cidABC* is activated in glucose-grown cells and in the presence of acetate [4,5]. Recently, it was revealed that CidA and CidB modulate cell death through the direct control of these overflow metabolic enzymes: CidC (pyruvate:menaquinone oxidoreductase) involved in acetate production and AlS (α-acetolactate synthetase) and AlD (α-acetolactate decarboxylase) involved in acetoin production [3]. This was a surprising conclusion, because Bayles and collaborators had previously hypothesized that CidA and a structural homolog named LrgA were functionally similar to bacteriophage holin/anti-holin proteins [6]. In this previous model, under stress conditions (such as acidification), the holin-like CidA may collapse the membrane potential and change the cell-wall pH, thus triggering cell-wall associated peptidoglycan hydrolases (PHs) activity and lysis [6]. According to Bayles, much about the Cid/Lrg family of cell-death modulators largely present in Gram positive and in Gram negative bacteria remains unknown; specifically the identification and characterization of PHs involved in this cell death phenomena remain largely unknown [7].

Recent work has found that YsbA protein -a LrgA homolog- is not involved in cell death control but in cell growth with pyruvate as the sole carbon source in *Bacillus subtilis* [8]. The *ysbAB* operon is highly expressed after glucose exhaustion and when *B*. *subtilis* grows in defined medium with pyruvate as only carbon source. In these growth conditions, the two-component system LytS/T is involved in the *ysbAB* expression while in the presence of glucose, the carbon regulator CcpA directly represses *ysbAB* expression [8].

The *Bacillus cereus* group includes *B*. *anthracis* and *B*. *cereus*, two well-known spore-forming pathogens of mammals [9]. The former is the organism that causes anthrax while the latter is frequently associated with food-borne infections that result in gastroenteritis [10]. These bacteria possess *cidAB*, *lrgAB* and *alsSD* operons and the *cidR* gene but not *cidC* [11]. Bayles and collaborators studied the CidR regulon in *B*. *anthracis* and found that in glucose-grown cells, the expression of the *cidAB*, *cidR*, and *alsSD* operons is activated in the late exponential phase [11]. They also showed that, as had been initially described in *S*. *aureus*, the CidR regulon is necessary for optimal survival in high-glucose grown cells at their late stationary phase of growth [11]. Despite this study, the functional link between the CidR regulon and glucose catabolism remains to be demonstrated, and the molecular basis for the role of CidAB in cell death is currently unknown in *B*. *anthracis*.

Members of the *B*. *cereus* group are unique in possessing two additional *cid* paralogs, the *clhAB*_*1*_ and *clhAB*_*2*_ operons, which have overall similarity scores that are >50% to the *cidAB* operon [12]. The *clhAB*_*2*_ operon encodes two putative integral membrane proteins, ClhA2 and ClhB2. In *B*. *anthracis*, expression of *clhAB*_*2*_ is repressed in glucose-grown cells (~0.6%) and a *clhAB*_*2*_-inactivated mutant presents complex phenotypes, including cell morphological and survival changes in the late stationary growth phase and reduced spore production [12]. The means by which expression of *clhAB*_*2*_ is regulated in *B*. *cereus* species and how it influences bacterial growth and sporulation are unknown.

Here, we investigated the expression and the physiological role of *clhAB*_*2*_ in *B*. *cereus* ATCC 14579 in the presence and absence of glucose. We first determined how *clhAB*_*2*_ responded to glucose signal and investigated the transcriptional regulators involved in the regulation of *clhAB*_*2*_ expression. Then, we constructed a *clhAB*_*2*_ deletion mutant and found that sporulation was not impaired, but *clhAB*_*2*_ mutant exhibited morphological changes during the late-exponential and early-transition growth phases. To analyse these morphological changes, the wild-type, the *clhAB*_*2*_ mutant and the complemented strains were imaged with FM4-64 dye and quantitative image analysis was performed. We found that cell shape, chain length and intra-chain cell arrangement of *clhAB*_*2*_ mutant were significantly different from wild-type chain in the presence but not in the absence of glucose.

## Materials and methods

### Bacterial strains, growth conditions, and glucose assay

*B*. *cereus* strains (Table 1) were grown at 37°C, with the exception of the growth rate analysis (see below), which was conducted at 30°C. Exponentially growing cultures of *B*. *cereus* were inoculated into standard LB medium [13] or LB supplemented with 0.35% glucose (LBG) at a final optical density (OD) of 0.05. Catabolyzable amino acids are plentiful in LB broth, presumably in the form of oligopeptides [13]. Glucose was added to LBG cultures at the onset of growth. The glucose concentrations in *B*. *cereus* cultures were determined using filtered supernatants and the Glucose (GO) Assay Kit (Sigma). Culture pH was monitored by pH electrode. With the exception of the growth rate analysis, all cultures were grown in flasks with an aeration ratio of 10 on a rotary shaker at 175 rpm. The onset of the transition growth phase (*t*_0_) was defined as the breakpoint in the slope of the log phase growth curve, and *t*_n_ is the number of hours before (-) or after time zero [14]. Quantitative image analyses were performed using *t*_0_ cultures and three independent cultures were performed for each experimental condition.

**Table 1:** Strains used.

Strain	Genotype	Source or reference
*B*.*cereus* ATCC 14579 (*Bc*)	Wild-type reference strain	[15,16]
Δ*clhAB*_*2*_	*Bc* Δ*clhAB*_*2*_::*tet*	This study
Δ*ccpA*	*Bc* Δ*ccpA*::*kana*	This study
Δ*codY*	*Bc* Δ*codY*	[16]
Δ*clhAB*_*2*_ Ω*clhAB*_*2*_	Δ*clhAB*_*2*_, pHT315 Ω*clhAB*_*2*_	This study
Δ*clhAB*_*2*_ *+*pHT315	Δ*clhAB*_*2*_, pHT315	This study
*clhAB*_*2*_’Z	*Bc*, pHT304-18Ω P_*clhAB2*_*’- lacZ* (locus tag BC5133-BC5132 = *clhAB*_*2*_)	This study
*clhAB*_*2*_’Z-Δ*ccpA*	Δ*ccpA*, pHT304-18 ΩP _*clhAB2*_*’*_*-*_ *lacZ*	This study
*clhAB*_*2*_’Z-Δ*ccpA-ccpA*	*clhAB*_*2*_’Z-Δ*ccpA*, Δ*amyE*:: *ccpA*	This study
*clhAB*_*2*_’Z-Δ*codY*	Δ*codY*, pHT304-18ΩP _*clhAB2*_*’*_*-*_ *lacZ*	This study
*clhAB*_*2*_’Z-Δ*codY* pHTcodY	Δ*codY*, pHT304-18Ω P_*clhAB2*_*’- lacZ* pHT1618KΩPxyl*codY*	This study
mCodYBox’Z	*Bc*, pHT304-18Ω PΔ_CodYBox_’-*lacZ*	This study
mCodYBox’Z-Δ*codY*	Δ*codY*, pHT304-18Ω PΔ_CodYBox_’-*lacZ*	This study

### Growth rate determination

Bacterial growth analysis was performed using a micro-plate reader system (Tecan Infinite F200PRO, Magellan software) with flat transparent 96-well plates (Greiner). The “GrowthRates v2.0” program was employed for growth rate determinations using output files of OD_610_ values from the microplate reader [17].

### Stationary phase survival and sporulation test

Viable cells were enumerated at the onset of the culture, *t*_0_, 24 h, 48 h, and 72 h using the serial dilution method. Sporulation test was performed as described in [14].

### DNA manipulation techniques

Chromosomal DNA was extracted from *B*. *cereus* cells with the Puregene DNA Purification kit (Gentra Systems, USA). Plasmid DNA was extracted from *E*. *coli* using QIAprep spin columns (QIAGEN, France). Restriction enzymes (New England Biolabs, USA) and T4 DNA ligase (New England Biolabs, USA) were used in accordance with manufacturer’s recommendations. Oligonucleotide primers were synthesized by Sigma-Proligo (Paris, France). PCR was performed in an Applied Biosystem 2720 Thermal cycler (Applied Biosystem, USA). Amplified fragments were purified with the QIAquick PCR Purification Kit (QIAGEN, France). Digested DNA fragments were extracted from gels with the QIAquick Gel Extraction Kit (QIAGEN, France). Nucleotide sequences were determined by Cogenics (Meylan, France).

### Construction of the *clhAB*_*2*_ deletion strain

The *B*. *cereus clhA*_*2*_ (BC5133) and *clhB*_*2*_ (BC5132) genes were deleted by homologous recombination, using the pRN5101 thermo-sensitive vector as previously described [18,19]. For this mutant construct, a tetracycline cassette was used for positive selection (Table 1). A fragment containing the 5’- and 3’- end flanking regions of the target gene and tetracycline cassette was inserted between the *Hind*III and *Bam*HI sites of pRN5101. Chromosomal allele exchange was confirmed by PCR with oligonucleotide primers located upstream of the 5’ fragment of the *clhAB*_*2*_ construct (clhA2Vf,5′-CGATAGGTGATTTGTGATAGG-3′) and downstream of the 3’ fragment (clhB2Vr,5′-CCGAAAGATAGGGGATGTA-3′).

### Construction of the *ccpA* deletion strain

For the *ccpA* mutant construct, a kanamycin cassette carrying the *aphA3* gene was used, and the overlapping PCR method was performed to construct the *ccpA*-mutated fragment (Table 1). This fragment, which contained the 5’- end flanking region of *ccpA*, the kanamycin cassette and the 3’- end flanking region of *ccpA*, was inserted between the *Eco*RI and *Bam*HI sites of the pMAD heat-sensitive vector [20]. Chromosomal allele exchange was confirmed by PCR with oligonucleotide primers located upstream of the 5’ fragment of the *ccpA* construct (ccpAVf, 5′- agtacatcccgatccagc -3′) and downstream of the 3’ fragment (ccpAVr, 5′- agttttcaacaaactaaca -3′).

### Plasmid construction

pHT304-P_*clhAB2*’_-*lacZ* was obtained by inserting the DNA region upstream (corresponding to the intergenic region) of the *B*. *cereus* ATCC 14579 *clhA*_*2*_ gene between the *Pst*I and *Xba*I cloning sites of pHT304-18Z using forward primer GM38 (5’-AAACTGCAG CACCACCTATCTTGTTTATCCCGTA-3′) and reverse primer GM39 (5’-GCTCTAGAGCATAATAGCAACGAGTGT-3′). The resulting plasmid was then transferred into *B*. *cereus* by electroporation.

Site-directed deletion of the CodY presumed box 5’-TAAATTCAGAAAATA-3’ was performed by PCR-driven overlap extension method [21] with mutagenic primers which share complementary sequence, F-codYBm (5'-TGAAATAATAGTCTTTAAAACTTTTTATATTAG) and R-codYBm (5'-CTAATATAAAAAGTTTTAAAGACTATTATTTCA) and flanking primers GM38 and GM39. pHT304-P_CodYBm_’-*lacZ* was obtained by inserting this mutated DNA fragment between the *Pst*I and *Xba*I cloning sites of pHT304-18Z and the resulting plasmid was then transferred into *B*. *cereus* by electroporation.

A *clhAB*_*2*_ complementation plasmid was constructed by amplifying a fragment (including the coding sequence of *clhA*_*2*_ and *clhB*_*2*_ and the promoter region) by PCR using the primer pair CAB2F (5′-GCGGATCCCCTATCTTGTTTATC-3′) and CAB2R (5′-CGGAATTCGCACTTGGCGTACCT-3′). The fragment was cloned in vector pHT315, which is presumably present at 15 copies per cell [22]. A *ccpA* complemented strain was constructed which harbored a copy of the *ccpA* gene integrated in the *amyE* locus. The *ccpA* gene was cloned in a modified pMAD thermo-sensitive vector [23], harboring upstream and downstream fragments of *amyE*. The recombinant plasmid was introduced into the *ccpA* mutant strain. Chromosomal allele exchange was confirmed by PCR. The pHT1618KΩPxyl*codY* strain was constructed in a previous analysis [24].

### β-Galactosidase assay

β-Galactosidase activity was assayed as described in [19].

### Fluorescence microscopy, image capture and analysis

The septum of dividing cells and cytoplasmic membranes were imaged using the FM4-64 lipophilic dye (Molecular Probes). Cells were isolated from cultures (see Growth conditions paragraph) and incubated with FM4-64 at 20 μg mL^-1^ at room temperature for 5 min. Slides were spotted with 4-μl aliquots, then cells were visualized by oil-immersion fluorescence microscopy (magnification of x100; NA of 1.4). Cell chains and individual cells were observed with a Zeiss Axio Observer.Z1 inverted fluorescence microscope equipped with a Zeiss AxioCam MRm digital camera and a custom-made filter (excitation: D510/40, beam splitter 540 DCLP, emission: D640/50). Fluorescent and phase-contrast images were processed with Zeiss ZEN 2–lite software.

#### Cell width determination

Using fluorescent digital images, cell width was measured from a traced line segment generated with the image analysis tools in FIJI software (see below for statistical analysis).

#### Determination of number of cells per chain

Using fluorescent digital images, the total number of cells per chain was counted using the “automatic particle counting” tool in FIJI software and was manually corrected.

#### Determination of short and long inter-constriction cell arrangements

Microscopy images of cell chains revealed strong constriction at some cell wall septa that connected cells within each chain. These deeper invaginations correspond to cells undergoing separation while other septa show no detectable constrictions. In our study, “short” inter-constriction arrangement contained 2–4 cells and “long” inter-constriction arrangement had 6–8 cells. Using fluorescent images, short and long cell arrangements were counted manually.

### Statistical analysis

#### Cell width measures

An ANOVA and a Student’s t-test analysis were performed using the means of each condition using R software (version 3.1.1) (R Core Team, 2014). These analyses examined three factors: day, mutation, and glucose. Our model took into account the main effects of these factors and the interaction effects between mutation and glucose. Between 270 and 320 measures were acquired for *Bc*, Δ*clhAB*_*2*_, and Δ*clhAB*_*2*_*ΩclhAB*_*2*_ in both growth conditions.

#### Determination of number of cells per chain

A non-parametric Mann-Whitney test and a Two-Sample Fisher-Pitman Permutation Test using R software (version 3.1.1) (R Core Team, 2014) were performed to examine differences in the number of cells per chain in the absence or presence of glucose for each strain. Between 90 and 130 chains were analyzed for *Bc*, Δ*clhAB*_*2*_, and Δ*clhAB*_*2*_*ΩclhAB*_*2*_ in both growth conditions.

#### Count of the occurrences of short and long inter-constriction cell arrangements

A quasibinomial generalized linear model (GLM) with overdispersion was used using R software (version 3.1.1, function = quasibinomial) (R Core Team, 2014). This analysis examined three factors: day, mutation, and glucose. Our model took into account the main effects of these factors and the interaction effects between mutation and glucose. Between 280 and 320 cell arrangements were counted for *Bc*, Δ*clhAB*_*2*_, and Δ*clhAB*_*2*_*ΩclhAB*_*2*_ in LB and in LBG media.

## Results

### Expression of *clhAB*_*2*_ in the presence or absence of glucose

**Fig 1:**
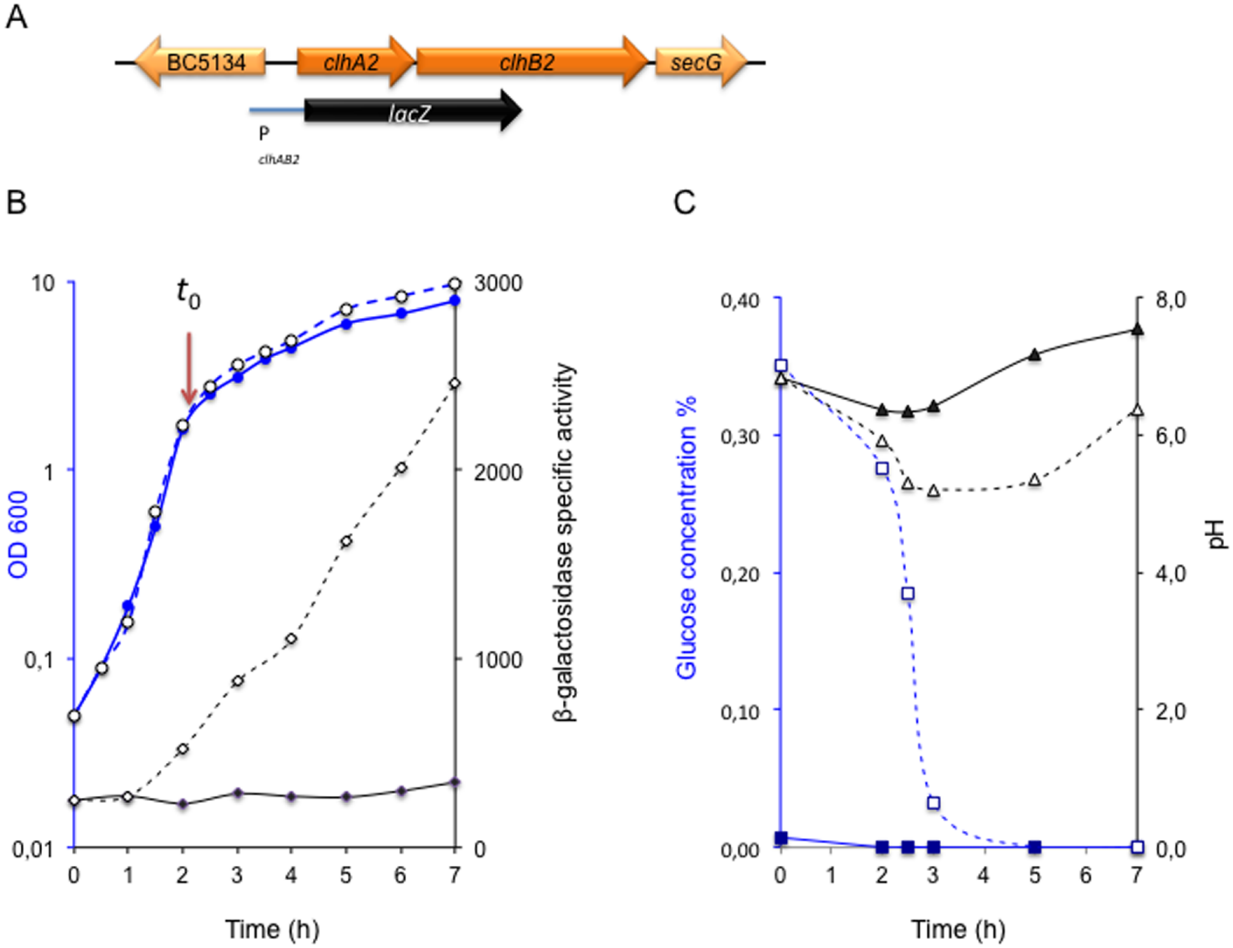
Expression of *clhAB*_*2*_ in the presence or presence of glucose. (A) Genetic organization of *clhAB*_*2*_ locus in the *Bc* genome and schematic representation of the *clhAB*_*2*_’Z transcriptional fusion construct. (B) *Bc* strain *clhAB*_*2*_’Z was grown in LB in the presence (open circles) or absence (closed circles) of 0.35% glucose. Optical densities (OD_600_, in blue circles) of cell cultures and β-galactosidase specific activities (U/mg protein, in black losanges) in the presence (open losanges) or absence (closed losanges) of 0.35% glucose are shown. The levels of *lacZ* expression of pHT304-18’Z (background level) were around 15 U/mg protein. (C) Filtered supernatants were measured for glucose concentration (Glucose Assay Kit, Sigma, in blue squares) and for pH determination (black triangles). The start of the transition growth phase is indicated as *t*_0_ for time zero. The glucose concentration of LB medium (closed squares) was below 0.01% and pH (closed triangles) was 7 ± 0.2. The data presented are representative of four independent experiments.

We first investigated how expression of the *clhAB*_*2*_ operon responded to the presence of glucose during growth of *B*. *cereus* ATCC 14579 wild-type strain (*Bc*). For this, a P_*clhAB2*_’-*lacZ* transcriptional fusion construct (Fig 1A) was introduced into the *Bc* strain, and the *clhAB*_*2*_’Z cells (log-phase cultures) were inoculated in LB [13] and LBG media. We quantified β-galactosidase activity from two hours before entry into the transition growth phase (*t*_0_, Materials and Methods) to five hours after (Fig 1B). We also measured glucose utilization along with changes in pH (Fig 1C). We observed that *clhAB*_*2*_ transcription remained at a constant low level in LB medium, but in the LBG medium, *clhAB*_*2*_ expression gradually increased from one hour before *t*_0_ to five hours after (Fig 1B). Following the increase in *clhAB*_*2*_ expression, glucose concentration decreased from 0.35% to 0.01% and pH values dropped from 6.8 to 5.2 (Fig 1C). The glucose concentration was estimated to be around 0.28% at the end of exponential growth phase and decreased below 0.03% one hour later. Remarkably, activation of *clhAB*_*2*_ expression was still observed despite the total glucose consumption. Furthermore, we observed similar patterns of *clhAB*_*2*_ expression when different initial concentrations of glucose (ranging from 0.35% to 1%) were used, as well as when cells were grown with fructose or sucrose instead of glucose (S5 Fig). Therefore, *clhAB*_*2*_ expression responded positively to the presence of glucose (as well as of two other rapidly fermented sugars), and this gene activation was concomitant with glucose consumption, decrease in pH, and entry into the transition growth phase.

In order to evaluate the influence of acidic pH on *clhAB*_*2*_ expression, we have used MOPS to buffer LB medium to grow *clhAB*_*2*_’Z cells. Again, we observed that *clhAB*_*2*_ transcription remained constant in LB-MOPS medium, but in the presence of glucose, *clhAB*_*2*_ expression gradually increased until *t*_1_ and then reached a plateau (S1 Fig). Thus, our results show that the presence of glucose but not acidic pH was involved in the activation of *clhAB*_*2*_ expression in our growth conditions.

### The regulation of the *clhAB*_*2*_ operon in the presence of glucose is CcpA-dependent

**Fig 2:**
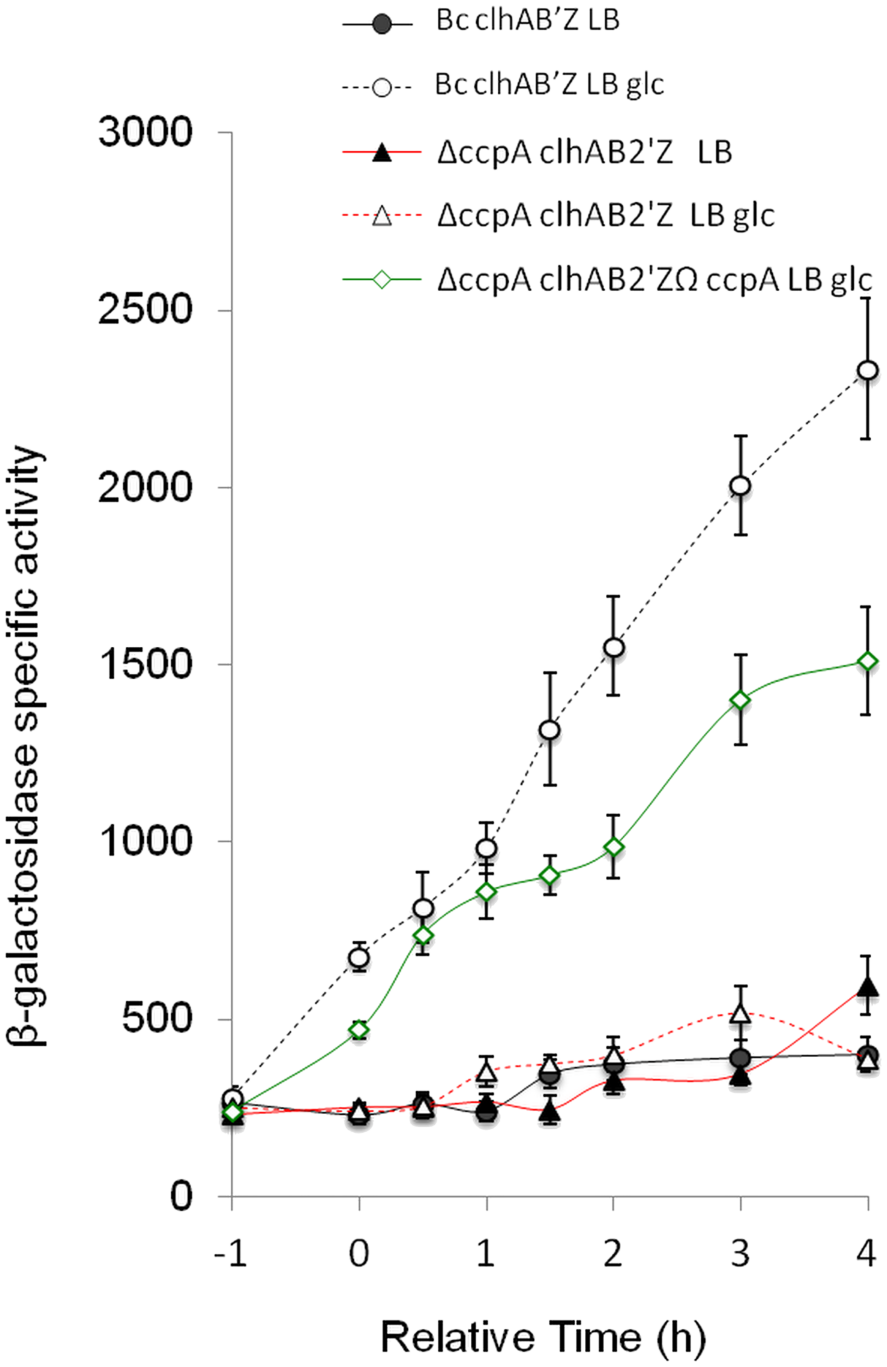
The glucose-activated expression of *clhAB*_*2*_ is abolished by the deletion of *ccpA*. Effect of *ccpA* mutation on the activation of *clhAB*_*2*_. Cells of *Bc* and isogenic mutant strains (Δ*ccpA*, *ccpA*-complemented mutant), which all harbored the transcriptional P_*clhAB2*_’-*lacZ* fusion construct, were grown in LB (closed symbols) or in LBG (open symbols) media. Samples were harvested at the indicated times and were assayed for β-galactosidase specific activity. Glucose 0.35% was added, when appropriate, at the onset of the culture. *t*_n_ is the number of hours before (-) or after *t*_0_. SD bars are shown.

In low G+C Gram-positive bacteria, transcription in response to the availability of a preferred carbon source such as glucose is mainly regulated by CcpA [25]. CcpA is a member of the LacI protein family of transcription factors, and can be either a positive or a negative regulator. CcpA is a DNA-binding protein that recognizes target promoters that contain the catabolite-responsive element (CRE) site, which is a 14-bp palindromic sequence [25]. We therefore decided to investigate the role of CcpA in the glucose-dependent activation of *clhAB*_*2*_ expression. A Δ*ccpA* mutant and a genetically complemented mutant were constructed, and the P_*clhAB2*’-_*lacZ* fusion was introduced in these mutant strains. Cells were grown in LB and LBG media, and β-galactosidase activity was measured one hour before entry into the transition growth phase (*t*_-1_) to four hours after (*t*_4_) (Fig 2). The deletion of *ccpA* abolished the glucose-induced activation of *clhAB*_*2*_ expression (Fig 2). Indeed, in the Δ*ccpA* mutant, *clhAB*_*2*_ expression in the presence of glucose was similar to that observed without glucose in the wild-type strain (Fig 2). The addition of *ccpA* at the *amy* chromosomal locus successfully complemented the Δ*ccpA* mutant such that β-galactosidase activity levels were actually similar to wild-type levels in LBG medium. This result demonstrated that CcpA control is required for glucose-activated *clhAB*_*2*_ expression. However, visual analysis of the *clhAB*_*2*_ promoter region did not identify any sequence that resembled the CcpA consensus sequence of *B*. *subtilis* or *B*. *cereus*, and a previous bioinformatic search had failed to identify a CRE motif in the promoter region of the *clhAB*_*2*_ gene [26]. We thus hypothesized that CcpA controls *clhAB*_*2*_ indirectly, through the altered expression or activity of other transcriptional regulators.

### CodY is needed for *clhAB*_*2*_ expression under all the conditions tested

CodY is a branched-chain amino acid and GTP sensor and a global regulator of transcription in low G + C Gram-positive bacteria [27,28]. CodY has been characterized in several bacterial pathogens [28–30] including *B*. *cereus* [16,24,31,32] and *B*. *anthracis* [33,34]. In *B*. *cereus* and *B*. *thuringiensis*, CodY is active under various rich laboratory media such as LB broth and BHI medium, during the exponential phase [16,24], as well during the early transition growth phase [16,24,32]. In *Bc* strain, numerous genes involved in biofilm formation, as well as amino acid transport and metabolism were upregulated, while genes associated with motility and virulence were repressed upon deletion of *codY* [16].

**Fig 3:**
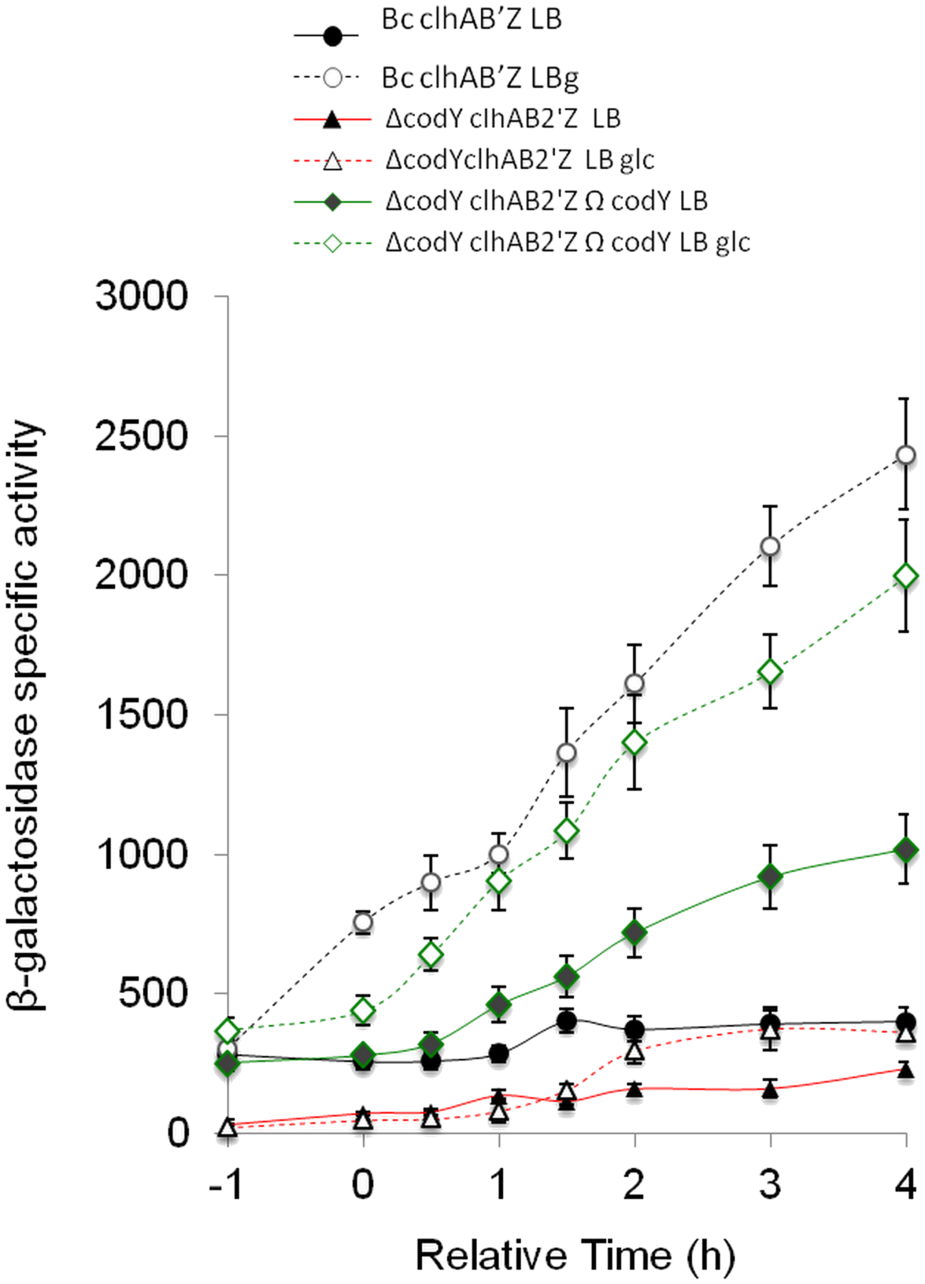
CodY-dependent regulation of the *clhAB*_*2*_ operon in the presence or absence of glucose. Effect of *codY* mutation on the expression of *clhAB*_*2*_. Cells of *Bc* and isogenic mutant strains (Δ*codY*, *codY*-complemented mutant), which all harbored the transcriptional P_*clhAB2*_’-*lacZ* fusion construct, were grown in LB (closed symbols) or in LBG (open symbols) media. See legend of the Fig 2 for additional informations.

We hypothesized that CodY could positively control *clhAB*_*2*_ expression under our nutrient-rich growth conditions. We measured the transcriptional activity of *clhAB*_*2*_ promoter fused to the reporter gene *lacZ* in wild-type and Δ*codY* cells, in LB and LBG media (Fig 3). The deletion of *codY* led to a strong decrease in β-galactosidase activity under all the conditions tested: the expression levels in the Δ*codY* mutant were very low (around 50–60 SA) until *t*_0.5;_ from *t*_0.5,_ in LB medium the expression levels were slightly increased (two- to threefold), and in LBG medium, the expression levels were gradually elevated (two- to eightfold); that said, all expression levels were still lower than the wild-type basal expression level (i.e., in LB medium). While complementation of the Δ*codY* mutant restored the *clhAB*_*2*_ expression under all the conditions tested, expression levels in LB medium were actually higher than those in the wild type (Fig 3). Our result demonstrated that CodY is active and needed for the transcription of the *clhAB*_*2*_ operon under all the conditions tested.

We also analyzed the CodY repressor activity in LB and LBG media during both the late exponential and transition growth phases until *t*_*4*_. We chose one of the most up-expressed genes in the *Bc* Δ*codY* mutant, namely BC2026, which encodes an OppA-like peptide binding and transport protein [16]. We measured the transcriptional activity of BC2026 promoter fused to the reporter gene *lacZ* in wild-type and Δ*codY* cells, in LB and LBG media (S2 Fig). As expected [16], the expression of P_*BC2026*_’-*lacZ* was abolished in *Bc* (around 10–15 SA, similar to background levels), while high expression levels (40- to 100-fold increase) were observed during exponential and transition growth phases until the end of the experiment (*t*_*4*_) in the *codY* mutant (S2 Fig). Again, this result demonstrated that CodY is clearly active under all the conditions tested.

### *clhAB*_*2*_ expression requires the presence of a 15-bp CodY-binding sequence with four mismatches

**Fig 4:**
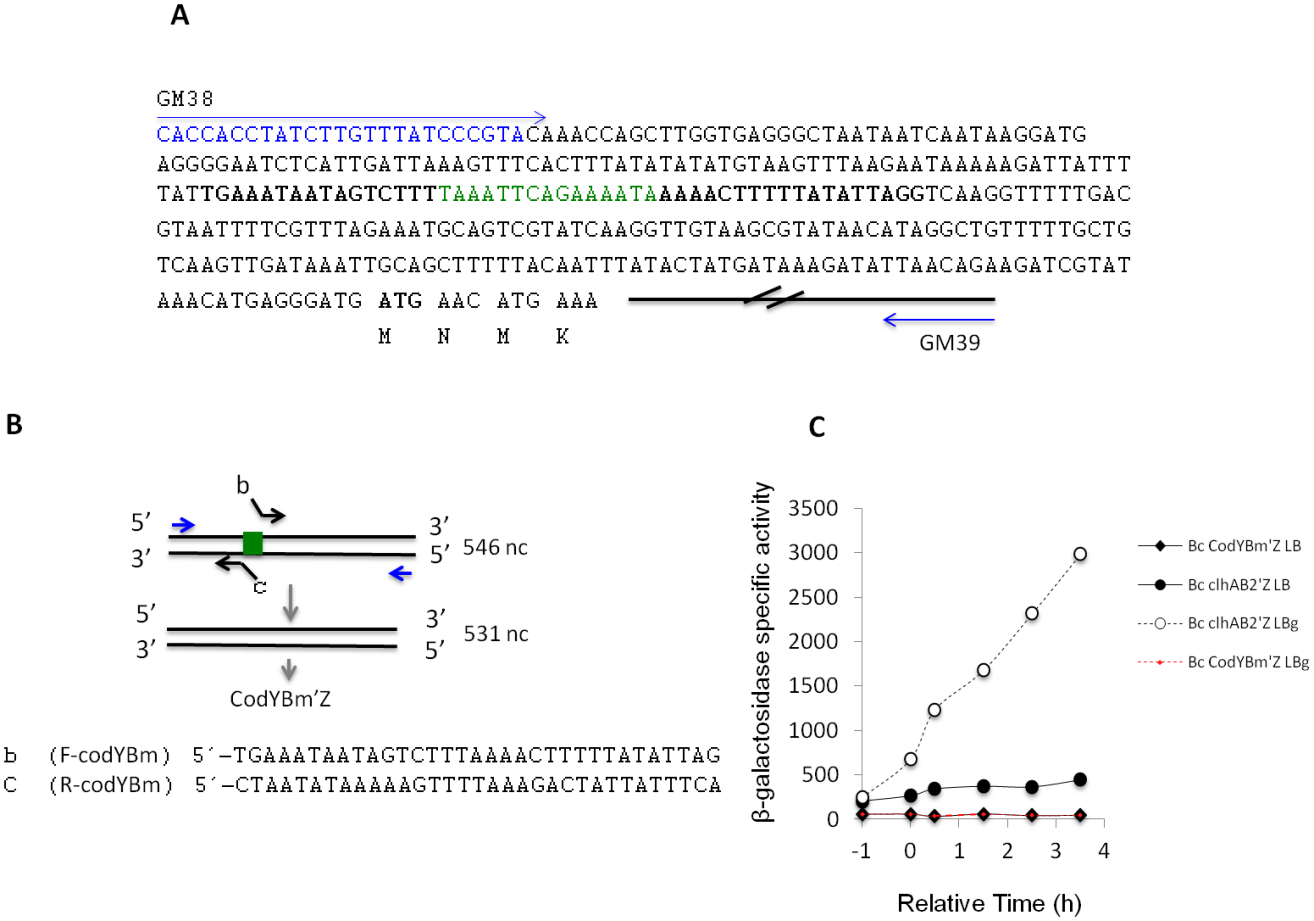
The *clhAB*_*2*_ expression requires the presence of a 15-bp putative CodY-binding motif. (A) The sequence of the *clhAB*_*2*_ regulatory region. GM38 primer (sequence in blue) and GM39 primer (see Materials and methods) used for the *clhAB*_*2*_-*lacZ* fusion construction are located. The CodY binding motif (in green) is identified 178 bp upstream from the likely initiation codon (in bold). Sequences used for SOE primers design are in bold. (B) Schematic drawing of the PCR-driven overlap extension method used for mutagenesis analysis. Primers sequences are shown. CodY binding motif, green box. (C) Effect of CodY binding motif deletion on *clhAB*_*2*_ expression. *Bc* cells harboring either P_*clhAB2*_’-*lacZ* fusion or P_CodYBm_’-*lacZ* fusion, were grown in LB (closed symbols) or LBG (open symbols) media. See legend of the Fig 2 for additional information. Representative experiment of n = 2 experiments are shown.

CodY is a unique DNA-binding protein that recognizes target promoters containing the CodY motif, which is a 15-bp consensus palindromic DNA sequence [35]. In *B*. *anthracis*, CodY target genes were identified by the genome-wide analysis of *in vitro* CodY-DNA complexes [36]. A CodY-binding fragment of 33-nt was identified upstream of the *B*. *anthracis clhAB*_*2*_ operon [36]. The CodY DNA-binding sequence, 5’-TAAATTCAGAAAATA-3’, which has four mismatches with respect to the CodY-binding consensus motif, AATTTTCWGAAAATT, was identified in this fragment [36]. We also found this CodY DNA-binding site sequence upstream of *clhAB*_*2*_, which was localized 178 bp upstream of the *Bc clhA*_*2*_ initiation codon (Fig 4A). We thus hypothesized that CodY directly activates *clhAB*_*2*_ through the DNA binding to this CodY motif.

To address the contribution of this CodY motif in *clhAB*_*2*_ expression, we deleted the 15-bp CodY motif sequence of the P_*clhAB2*_ insert, resulting in the fusion of P_CodYBm’_-*lacZ* (see Materials and methods and Fig 4B). The expression of the P_CodYBm’_-*lacZ* fusion was abolished under all the conditions tested (around 10–15 SA, similar to background levels) (Fig 4C). Thus, our results showed that *clhAB*_*2*_ expression absolutely requires the presence of this 15-nt sequence under all the tested conditions. The P_CodYBm’_-*lacZ* fusion was also introduced into the Δ*codY* mutant, and cells were grown in LB and LBG media. Again, the expression of the P_CodYBm’_-*lacZ* fusion was abolished under all the tested conditions. Overall, our results suggested that *clhAB*_*2*_ expression requires the presence of this 15-nt sequence.

### The deletion of *clhAB*_*2*_ did not affect growth and sporulation in *B*. *cereus* ATCC 14579

**Fig 5:**
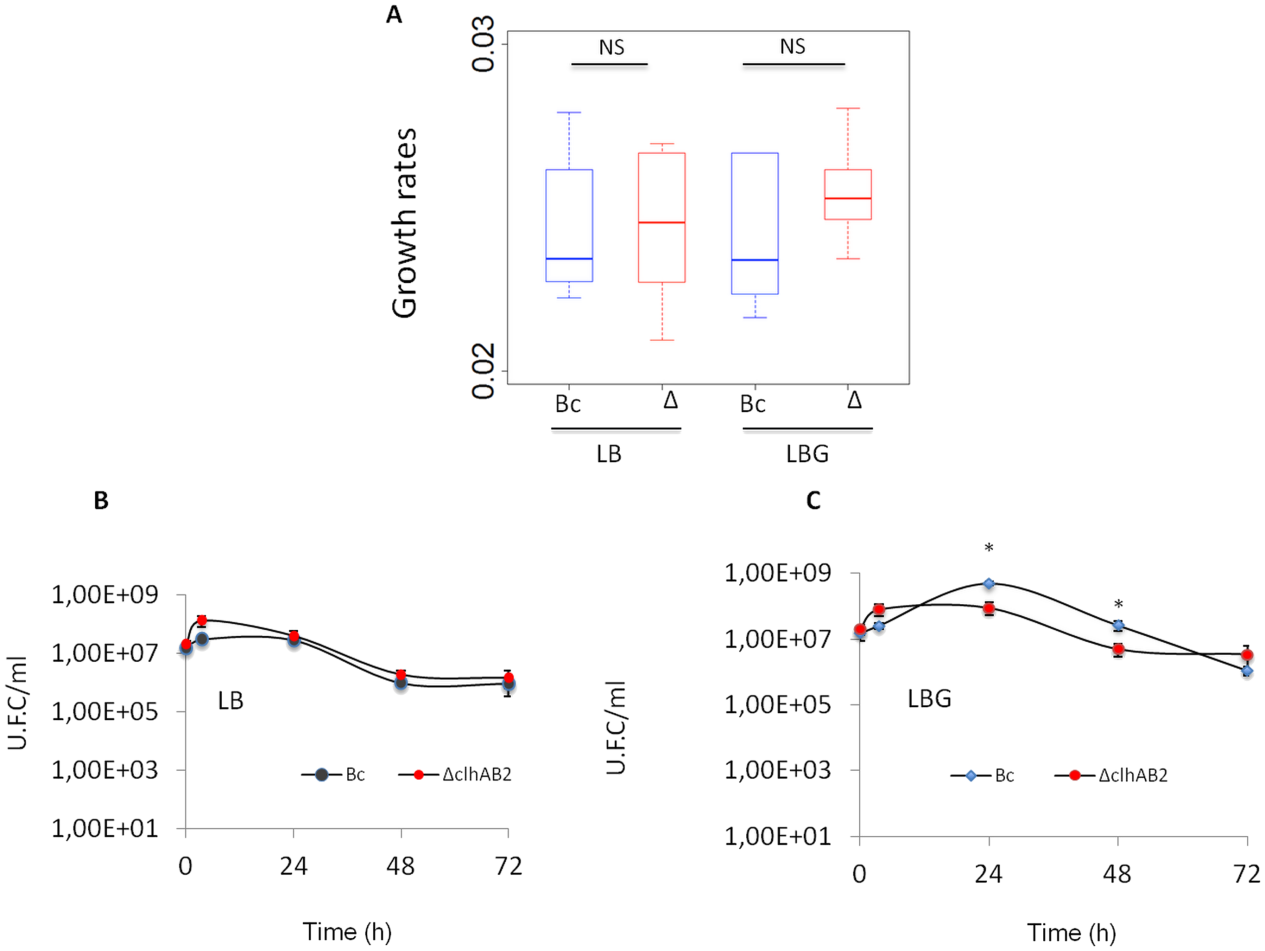
Phenotypic analyses of Δ*clhAB*_*2*_ isogenic mutant. (A) Box plot of growth rates in the absence (LB) or presence of 0.35% glucose (LBG). Growth rates were determined using output files of OD_610_ values from the microplate reader (see Materials & Methods). NS—no statistical significance. (B, C) Stationary phase survival of *Bc* and Δ*clhAB*_*2*_ isogenic mutant in LB and in LBG. Cell viability tests were performed at 0, 4, 24, 48, and 72h. Significance is based on Mann & Whitney test with a *P* <0.05*.

We investigated the role of the *clhAB*_*2*_ operon in the *Bc* wild-type strain by deleting the entire operon via allelic exchange. *Bc* and Δ*clhAB*_*2*_ strains grew similarly, with comparable growth rates in LB and LBG media (Fig 5A). This finding suggested that *clhAB*_*2*_ is not necessary for exponential growth. We also assessed the Δ*clhAB*_*2*_ mutant in terms of cell viability in the absence (Fig 5B) or presence (Fig 5C) of glucose after 24, 48, and 72 hours of incubation. Small significant differences (*P*<0.05) were observed between Δ*clhAB*_*2*_ and *Bc* in stationary phase survival after 24 and 48 hours of growth in LBG **(**Fig 5C). Spore production was performed in a specific sporulation medium, but no significant differences were observed in sporulation tests. Indeed, the median spore production of *Bc* at 72 h was 1.3·10^8^ spores/ml (min = 7.2·10^7^; max = 1.9·10^8^, n = 5), while that of the Δ*clhAB*_*2*_ mutant was 9.5·10^7^ spores/ml (min = 6.1·10^7^; max = 1.7·10^8^, n = 5). The fact that the Δ*clhAB*_*2*_ mutant did not exhibit reduced sporulation efficiency compared to wild-type stands in contrast to report from *B*. *anthracis* [12] and suggests the existence of differences in the sporulation process of these two bacteria.

### *clhAB*_*2*_ is required for chain lengthening in the presence of glucose

**Fig 6:**
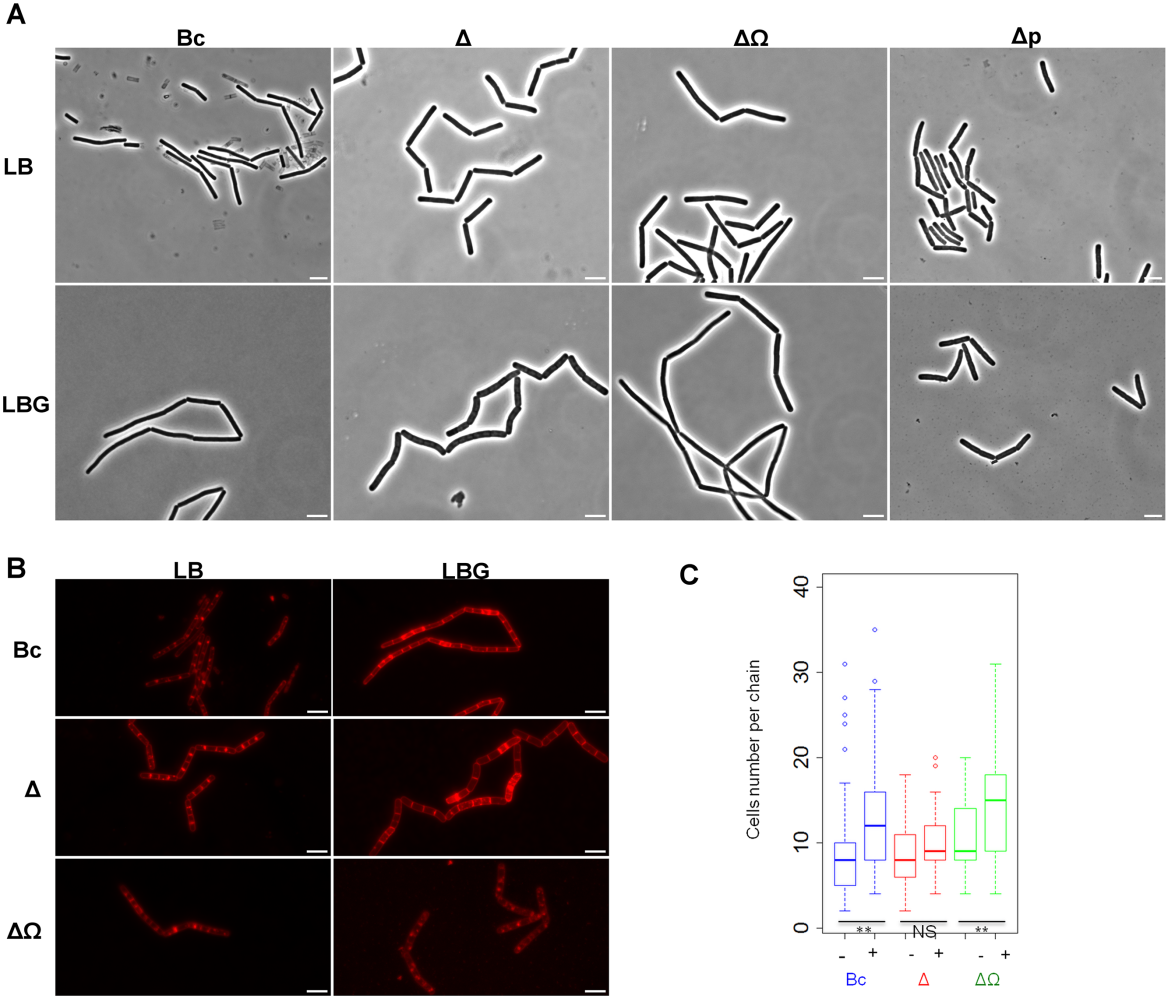
Chain lengths in *Bc*, Δ*clhAB*_*2*_, and Δ*clhAB*_*2*_*ΩclhAB*_*2*_ populations in the presence or absence of glucose. (A) Phase-contrast images of cell-chains at *t*_0_ of wild-type (*Bc*), Δ*clhAB*_*2*_ (Δ), complemented (ΔΩ) and pHT315 (Δp) mutant strains grown in LB medium and LB medium supplemented with glucose (LBG). Images of chains revealed strong constrictions (deeper invaginations) corresponding to cells undergoing separation. Scale bar (5μm) is shown for each image. (B) Fluorescent micrographs of *Bc*, Δ, and ΔΩ cell-chains at *t*_0_ in LB and LBG. Division septa and cytoplasmic membranes were imaged using the FM4-64 lipophilic dye. Scale bar (5μm) is shown for each image. (C) Box plots of chain length (number of cells per chain) at *t*_0_ in *Bc* (blue), Δ*clhAB*_*2*_ (red), Δ*clhAB*_*2*_Ω*clhAB*_*2*_ (green) populations. Between 90 and 130 chains from at least three independent cultures were analysed. No fewer than 1,000 cells were quantitated for each strain represented in the graph using fluorescence micrographs (see Materials and methods). Median (strong line in the box), interquartile range (IQR; box), whiskers (1.5 x IQR) and outliers (dot) are presented. Significance is based on two tests, Mann-Whitney and Two-Sample Fisher-Pitman Permutation, with a *P* of <0.01**. Non significative (NS), *P*> 0.05.

**Fig 7:**
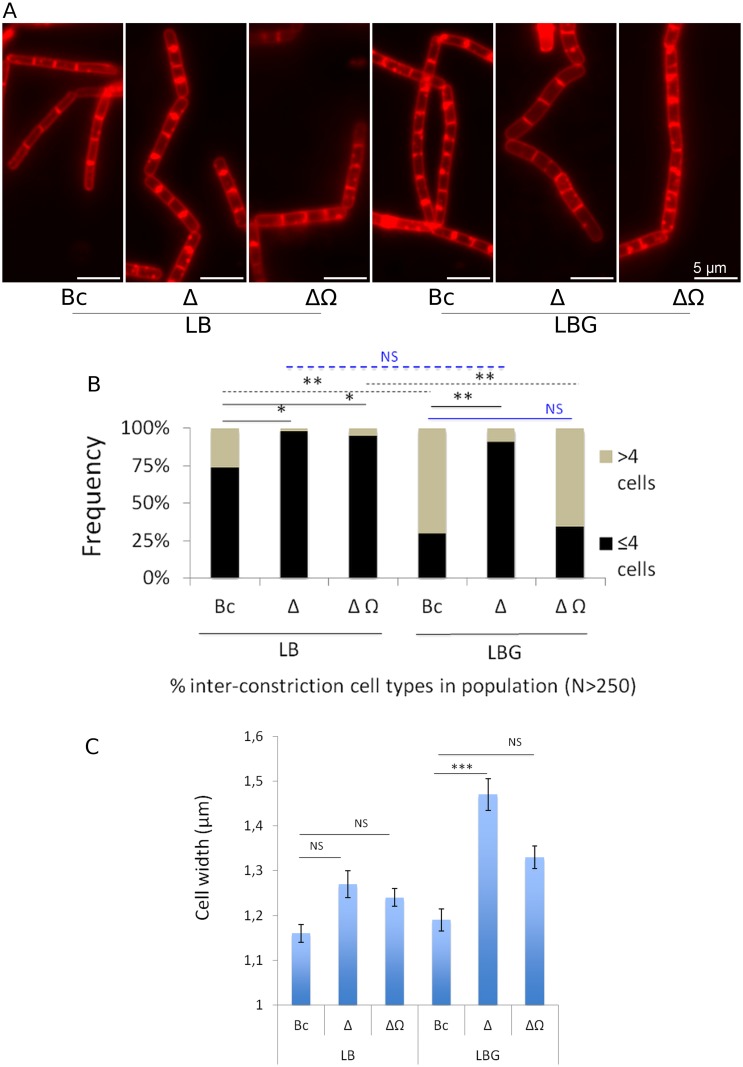
Inter-constriction cell arrangements and cell width measures in *Bc*, Δ*clhAB*_*2*_, and Δ*clhAB2ΩclhAB*_*2*_ populations in the presence or absence of glucose. (A) Close view of *Bc*, Δ*clhAB2*, and Δ*clhAB2*,*ΩclhAB2* chains at *t*_0_ using fluorescence microscopy. Cells were grown in LB and LB with 0.35% glucose (LBG). Division septa and cytoplasmic membranes were imaged using the FM4-64 lipophilic dye. Chains exhibited constrictions that occurred at septa spaced 4 cells apart in *Bc*, Δ*clhAB*_*2*,_ and Δ*clhAB*_*2*_*ΩclhAB*_*2*_ in LB medium and in Δ*clhAB*_*2*_ in LBG medium. They also exhibited constrictions that occurred at septa spaced 8 cells apart in *Bc* and Δ*clhAB*_*2*,_Ω*clhAB*_*2*_ in LBG medium. (B) Distributions of “short” (≤4) and “long” (>4) inter-constriction cell types in the *Bc*, Δ*clhAB*_*2*_, and complemented mutant populations (N>250 cell arrangements). Two inter-constriction arrangement types in *Bc*, Δ*clhAB*_*2*_, and Δ*clhAB*_*2*_Ω*clhAB*_*2*_ populations were defined (see Materials and methods). The first type, containing cell arrangements with two to four cells ("short") and the second type, including cell arrangements with six to eight cells ("long"). The significant effects of glucose (dashed line) and *clhAB*_*2*_ mutation (solid line) are based on a Binomial analysis (see Materials and methods) with *P* <0.01** and <0.05*. (C) Cell width measures in *Bc*, Δ*clhAB*_*2*_ and Δ*clhAB*_*2*_Ω*clhAB*_*2*_ populations (N>250 bacilli) in LB and LBG media. The significant effect of *clhAB*_*2*_ mutation in LBG medium is based on a Student’s t test and ANOVA (see Materials and methods) with *P* <0.001***. A *P* value close to the cutoff 0.05 was considered as non significant (NS). Mean ± CI _95%_ is depicted.

In bacteria of the *B*. *cereus* species, the formation of rod-shaped cell chains of different lengths appears to be a normal aspect of growth in different media and environments [37–39]. We observed that the *Bc* wild-type strain (*Bc*) produced short chains in LB medium and long chains and occasionnally serpentine chains in LBG medium during exponential (*t*_-1_, S3 Fig) and early transition growth (*t*_0_, Fig 6A; *t*_2_, S3 Fig). Instead, during the same growth period, the Δ*clhAB*_*2*_ mutant bacteria grew as a population of short chains in both growth conditions; in LBG medium, chains were wide and occasionnally curved (Fig 6A, S3 Fig). The complemented Δ*clhAB*_*2*_ strain carrying the *clhAB*_*2*_ operon on a plasmid showed an absence of this mutant chaining phenotype while the *clhAB*_*2*_ strain carrying the empty plasmid showed the mutant chaining phenotype in LBG medium (Fig 6A). To further analyze changes in chain morphology, fluorescent images of cell membranes and septa were examined at the onset of the transition phase (*t*_*0*_; Fig 6B). Aberrant septa locations were never observed in Δ*clhAB*_*2*_ cells (Figs 6B and 7A), suggesting that the *clhAB*_*2*_ operon is not involved in cell division. In addition, both wild-type and Δ*clhAB*_*2*_ mutant chains exhibit peritrichous flagella using transmission electronic microscopy, suggesting that the *clhAB*_*2*_ mutation did not alter neither the structure nor the implantation of flagella (S4 Fig).

We quantified the total number of cells per chain in *Bc*, Δ*clhAB*_*2*_, and Δ*clhAB*_*2*_Ω*clhAB*_*2*_ populations in the absence and presence of glucose, and displayed these data as box-and-whisker plots in order to show the shape of the distribution, its central value and the variability of the chain length (Fig 6C). *Bc* chains lengthened significantly in the presence of glucose (*P*<0.01). The median chain length of *Bc* in LB medium was 8 cells per chain and in LBG medium was 12 cells (interquartile ranges (IQRs); LB vs. LBG, 5 vs. 8 cells). In sharp contrast, glucose-induced chain lengthening was not observed in the Δ*clhAB*_*2*_ population: the median chain length of Δ*clhAB*_*2*_ was 8 and 9 cells per chain respectively in LB and LBG media, with remarkably small dispersion in LBG media (IQRs: LB vs. LBG, 5 vs. 4 cells). Δ*clhAB*_*2*_Ω*clhAB*_*2*_ chains lengthened significantly in the presence of glucose (*P*<0.01), demonstrating that the expression of *clhAB*_*2*_ in trans was sufficient for restoration of the wild-type chaining phenotype. The median chain length of Δ*clhAB*_*2*_Ω*clhAB*_*2*_ strain in LB medium was 9 cells per chain (IQR: 6) and in LBG medium was 15 cells (IQR: 9) (Fig 6C). This quantitative analysis revealed that *Bc* chains lengthened in the presence of glucose and *clhAB*_*2*_ is required for glucose-dependent chain lengthening. In other words, our data showed that *clhAB*_*2*_ down-regulates cell separation process during chain production in glucose-grown cells.

### *clhAB*_*2*_ is required for long inter-constriction cell arrangement in the presence of glucose

Microscopy images of wild-type, Δ*clhAB*_*2*,_ and Δ*clhAB*_*2*_Ω*clhAB*_*2*_ chains revealed deep invaginations or constrictions at cell wall septum connecting cells undergoing separation (Fig 6A and 6B). We repeatedly observed in LB medium that constrictions occurred frequently at septa that were spaced 4 cells apart in *Bc*, Δ*clhAB*_*2*_, and Δ*clhAB*_*2*_Ω*clhAB*_*2*_ populations (Fig 7A). The frequency of 4-chained-cell arrangements in *Bc*, Δ*clhAB*_*2*_, and Δ*clhAB*_*2*_Ω*clhAB*_*2*_ populations were similar (56, 52, and 57%, respectively, N = 150 cell arrangements, see Materials and methods). In LBG medium, we were intrigued by two observations: the occurrence of 4-cell arrangements decreased compared to that found in LB medium, and constrictions also often occurred at septa that were spaced 8 cells apart in the *Bc* population (Fig 7A). In *Bc*, the frequencies of 4- and 8-cell arrangements were 29% and 23%, respectively in LBG medium (N = 150, see Materials and methods). Instead, in the Δ*clhAB*_*2*_ population, the frequency of the 4-cell arrangement did not decrease (48%) and the 8-cell arrangement was observed at a very low frequency (4%) compared with that found in *Bc*. In Δ*clhAB*_*2*_Ω*clhAB*_*2*_ the frequencies of 4- and 8-cell arrangements were 31% and 35%, respectively, demonstrating that *clhAB*_*2*_ expression was sufficient for restoration of the wild-type inter-constriction cell arrangement pattern.

In order to have a meaningful picture of these inter-constriction cell arrangements in both growth conditions, we examined the distribution of “short” (≤ 4 chained cells) versus “long” (> 4 chained cells) cell arrangements in a larger *Bc*, Δ*clhAB*_*2*_, and Δ*clhAB*_*2*_Ω*clhAB*_*2*_ populations (N>250 cell arrangements) at the onset of transition phase (*t*_0_) (Fig 7B). In the absence of glucose, the percentages of short and long cell arrangements were 74% and 26%, respectively, but when glucose was present in the medium, these numbers swapped, to 30% short and 70% long (*P*<0.01) (Fig 7B). In sharp contrast, glucose-induced long cell arrangement was not observed in the Δ*clhAB*_*2*_ population. In the absence of glucose, the percentages of short and long cell arrangements were 98% and 2%, respectively, and when glucose was present, these numbers were not different (91% short and 9% long) (*P*>0.05) (Fig 7B). The Δ*clhAB*_*2*_ distribution of short and long cell arrangements in the presence of glucose was significantly different from the wild-type distribution (*P*<0.01) (Fig 7B). A *clhAB*_*2*_ mutant produced short cell arrangement in the presence of glucose. Glucose also had a remarkable effect on intra-chain cell arrangement in the Δ*clhAB*_2,_Ω*clhAB*_2_ population: in the absence of glucose, the percentages of short and long intra-chain arrangements were 95% and 5%, respectively, but when glucose was present in the medium, these numbers swapped, to 34% short and 66% long (*P*<0.01) (Fig 7B). Our results suggested that the expression of *clhAB*_*2*_ in trans was sufficient for restoration of *Bc* inter-constrictions cell arrangement in LBG medium (Fig 7B). Thus, *clhAB*_*2*_ is required for long inter-constriction cell arrangement in the presence of glucose. Overall, we demonstrated that *Bc* cells form short chains mainly associated with short cell arrangement (74%) in LB medium, but in the presence of glucose, *Bc* cells form significantly longer chains that are mainly associated with long cell arrangement (70%).

### *clhAB*_*2*_ mutation produced wide cells in the presence of glucose

The cell wall has multiple functions during bacterial growth, including maintaining bacterial cell integrity and shape by resisting internal turgor pressure. Compared with other Gram-positive rod-shaped bacteria, *B*. *cereus* is large (1.0–1.2 μm by 3.0–5.0 μm) [10]. In LB medium, we observed a small but not significant enlargement in Δ*clhAB*_*2*_ cells (Fig 5C). In LBG medium, Δ*clhAB*_*2*_ cells were significantly wider (*P*< 0.001) than wild-type cells (1.47 μM ± 0.04 CI _95%_ wide vs. 1.19 μM ± 0.03 CI _95%_ for *Bc*) (Fig 5C). Then, we showed that the expression of *clhAB*_*2*_ in trans was partially sufficient for restoration of *Bc* width (1.33 μm ± 0.04 CI _95%)_ in LBG medium. Overall, these data indicated that *clhAB*_*2*_ cells exhibited a decrease in cell wall resistance to the internal turgor pressure in LBG medium.

### Autolytic behavior of Δ*clhAB*_*2*_

**Fig 8:**
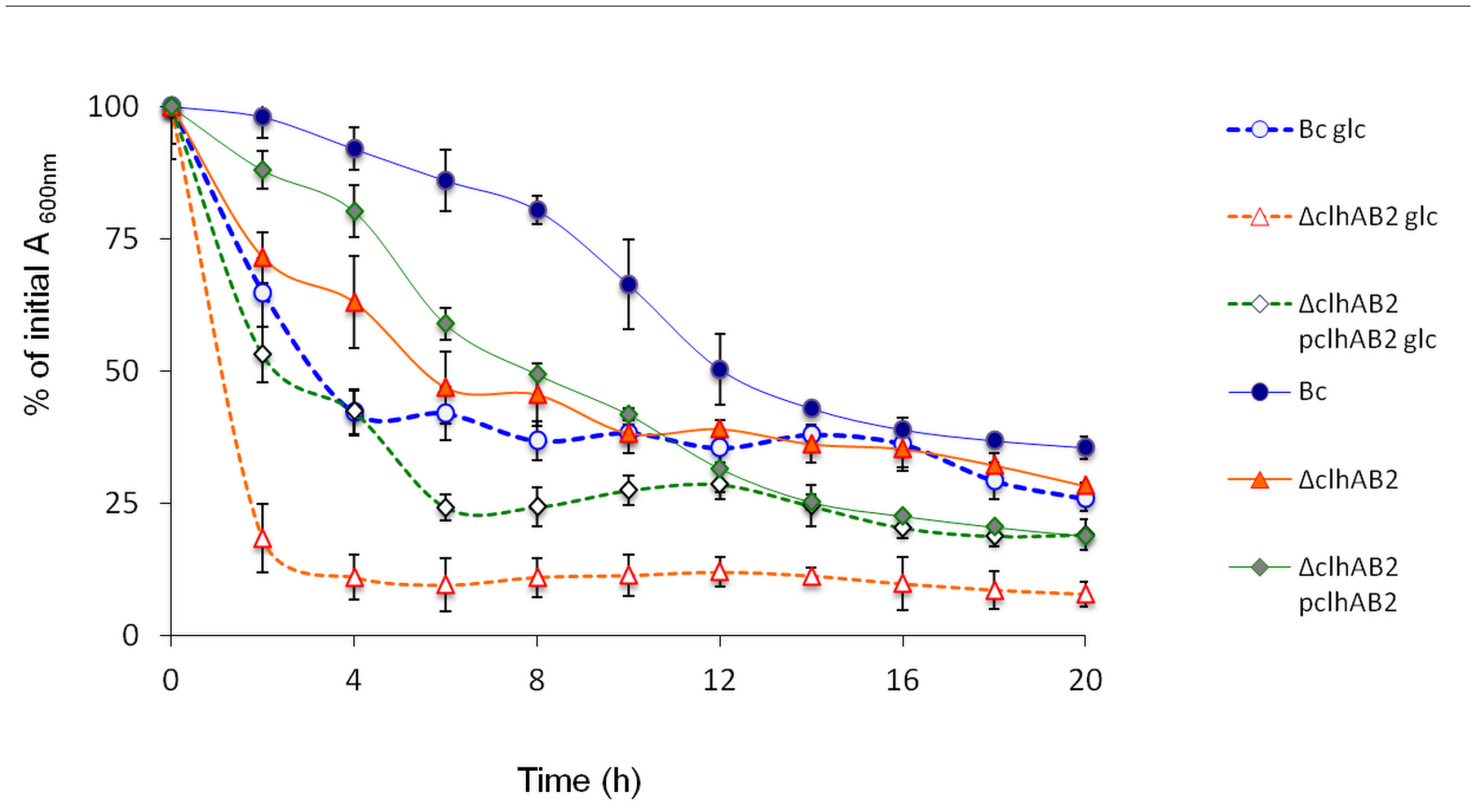
Autolysis of *B*. *cereus* wild-type ATCC 14579 (Bc), Δ*clhAB*_*2*_ mutant (Δ), complemented mutant (ΔΩ) strains in PBS buffer. The autolytic rate was expressed as the percentage decrease in the OD_610_nm. Mean values (SD, n = 4). % of OD values of the Δ*clhAB*_***2***_ are all significant different from *Bc* between 2 and 20h in LBG and in LB between 6 and 12h. Significance is based on Mann & Whitney test with a P-value of <0.01**. Symbols. *Bc* (blue circle), Δ *clhAB*_*2*_ (orange triangle) and the Δ*clhAB*_*2*_Ω *clhAB*_*2*_ (green losange).

Certain PHs, called autolysins, destroy the peptidoglycan mesh by cleaving peptidoglycan glycan strands or cross-links of the producer strain, resulting in cell lysis [6,40]. We lastly characterized the autolytic behavior of Δ*clhAB*_*2*_ cells by analysing the autolytic rate. As *B*. *cereus* demonstrates a high degree of autolysis around neutral pH [41], we analyzed autolysis in PBS buffer using late exponential cells, grown in LB or LBG media (Fig 8). The Δ*clhAB*_*2*_ strain showed accelerated autolysis compared to its parent strain in both media, which suggests again that PH activity is deregulated in the mutant. In addition, glucose had a remarkable effect on the observed differences (Fig 8). After four hours of incubation, the Δ*clhAB*_*2*_ autolysis rate was 40%, compared with 10% for *Bc*. Instead, with glucose-grown cells, the Δ*clhAB*_*2*_ autolysis rate was surprisingly high, 90% versus 60% for *Bc*. Furthermore, the autolysis rates of *Bc* and the Δ*clhAB*_*2*_Ω*clhAB*_*2*_ complemented strain were not different in either medium after four hours of incubation. Our results suggested that the expression of *clhAB*_*2*_ in trans was sufficient for restoration of *Bc* autolysis in both growth conditions. Overall, these data indicate that *clhAB*_*2*_ negatively controlled autolysis phenomena in *B*. *cereus*, while glucose appears to enhance autolysis phenomena.

## Discussion

### Roles of CodY and CcpA in the regulation of *clhAB*_*2*_ operon

Although the Cid/Lrg regulation network has been extensively analyzed in *S*. *aureus*, only a few studies have been performed to identify the expression and function of these genes in other bacterial species. The present study aims to investigate the expression and function of the *Bc clhAB*_*2*_ operon which is a *cid/lrg* homolog, during bacterial growth in a nutrient-rich medium with or without glucose. Here, we show that the global transcriptional regulatory protein CodY is required for the basal level of *clhAB*_*2*_ expression under all conditions tested while CcpA, the major global carbon regulator, is needed for the high-level expression of *clhAB*_*2*_ in glucose-grown cells. Our genetic evidence suggests that CcpA control is exerted in an indirect way in the presence of glucose (i.e late-exponential growth phase) and the regulatory pathway remains to be characterized. In *B*. *subtilis*, the CcpA network analysis remains complex as CcpA controls a high number of regulators directly (i.e regulation of gene expression) or indirectly (i.e modulation of activity) [25,42,43]. In *B*. *cereus*, the transcriptome analysis of the *ccpA* mutant showed that CcpA positively and negatively regulates several putative transcriptional regulators suggesting that the CcpA regulatory network is also complex in *B*. *cereus* [26].

Next, we further analyzed the known *in vitro* CodY-binding sequence (5’-TAAATTCAGAAAATA-3’)[36] localized upstream of *clhAB*_*2*_. We selected a straightforward mutagenesis analysis, in which the total deletion of the CodY-motif sequence was performed. Our results showed that this 15 nt-sequence is absolutely required for mediating the *clhAB*_*2*_ expression, as the deletion of this sequence abolished the *clhAB*_*2*_ expression. However, we are aware that this CodY-binding site could be located immediately upstream of the -35 region or overlap with the *clhAB*_*2*_ promoter. More functional analyses of the CodY-binding motif by point mutational analysis, together with the determination of the transcriptional start of *clhAB*_*2*_, should enable one to conclude that CodY activates the expression of *clhAB*_*2*_ through DNA binding to this CodY motif.

### Model of *clhAB*_*2*_ regulation in the presence or absence of glucose

**Fig 9:**
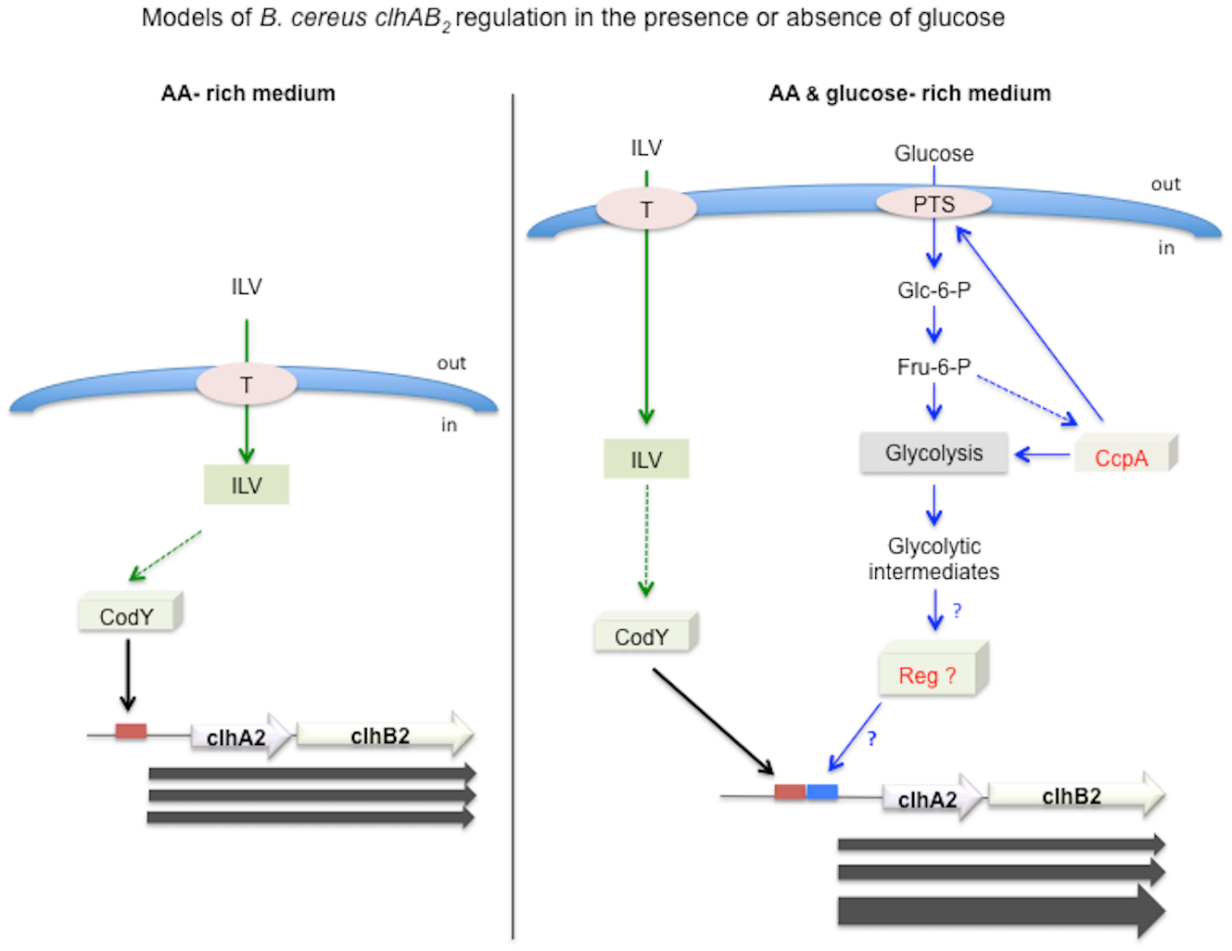
Two models for *B*. *cereus clhAB*_*2*_ regulation. Left: regulation of *clhAB*_*2*_ expression in an amino acid- rich medium. In a nutrient-rich medium (such as LB medium [13]), ILV uptake is sufficient to maintain the endogenous pool of ILV. The CodY global regulatory protein displays enhanced affinity for its DNA target when bound to ILV[27]. ILV-bound CodY binds to the CodY binding sequence upstream of *clhAB*_*2*_ and assists RNA polymerase with transcribing the *clhAB*_*2*_ operon (Figs 3 and 4). The expression level of *clhAB*_*2*_ is constant and moderate (i.e. basal level) during the late exponential and transition growth phases (Fig 1). Right: regulation of *clhAB*_*2*_ expression in an amino acid- glucose- rich medium. In LB medium with 0.35% glucose, ILV-CodY binds to the CodY motif and activates *clhAB*_*2*_ expression, but the expression profile is different (Fig 1). CcpA plays a positive role by indirectly regulating the transcription of *clhAB*_*2*_ (Fig 2) and this regulatory pathway remains to be characterized. The role of known or hypothetical effector molecules is depicted with a dashed arrow. In the left part, the ILV effector [27] is depicted with a green dashed arrow; in right part, the Fru-6-P [25] and unknown glycolytic intermediate effectors are depicted with blue dashed arrows. Moderate and constant *clhAB*_*2*_ expression is depicted by three identical gray arrows. High and gradual *clhAB*_*2*_ expression is depicted by three non-identical gray arrows. The intracellular ILV pool is depicted as a green box. The CodY motif sequence is depicted as a red box. The unknown DNA-binding motif is depicted as a blue box.?, unknown CcpA-dependent signaling pathway; ?, unknown transcriptional regulator. Fru-6-P, fructose-6-phosphate, Glc-6-P, glucose-6-phosphate. ILV, isoleucine, leucine, valine. CodY, CodY transcriptional regulator. CcpA, CcpA global carbon regulator; PTS, phosphotransferase system. T, unknown transporter of ILV.

The model in Fig 9 depicts the regulation of *clhAB*_*2*_ expression in LB medium and in LB medium with glucose. This model is based on our results, as well as on *B*. *subtilis* and *B*. *cereus* studies [16,25–27,30,35]. In this regulatory model, we assume that CodY protein binds specifically to the 15-nc CodY motif localized upstream of *clhAB*_*2*_ (Fig 4). When *Bc* cells grow in a nutrient-rich medium such as LB medium, the uptake of exogenous ILV is enough to maintain ILV homeostasis. CodY integrates the ILV signal as ILV is a major effector molecule of CodY protein. ILV-bound CodY binds to the CodY binding sequence and assists RNA polymerase with transcribing the *clhAB*_*2*_ operon. When glucose is available, CcpA integrates the glucose signal through the control of glucose uptake (via the PTS system) and the control of glycolysis. In parallel, CodY integrates the ILV signal. As discussed above, it remains unclear how CcpA controls *clhAB*_*2*_ expression in the presence of glucose. CcpA could control directly or indirectly an additional transcriptional regulator directly involved in the transcription of *clhAB*_*2*_ operon.

Is there a regulatory link between the CcpA and CodY signaling pathways in the regulation of *clhAB*_*2*_ expression? The answer will require further analyses, which are beyond the scope of this study. Interconnections between the CcpA and CodY regulons in carbon and nitrogen metabolism have been well documented in *B*. *subtilis* [43–46], but these connections have not yet been addressed in detail in the *B*. *cereus* group. The fact that *clhAB*_*2*_ expression is controlled by such important major regulators provides evidence of the important role of *clhAB*_*2*_ products in the physiology of *B*. *cereus*.

### Role of *clhAB*_*2*_ in the regulation of peptidoglycan hydrolase activity

The *Bc* genome presents 42 putative and largely unknown peptidoglycan hydrolases (PHs) [47]. PHs are involved in fundamental aspects of bacterial physiology: peptidoglycan growth, daughter cell separation during cell division and remodeling of the peptidoglycan sacculus to determine cell shape [40,48,49]. In addition, some PHs are members of the bacterial autolytic system involved in cell death phenomena (autolysis)[40]. Furthermore, bacteria in general can have a large number of PHs with redundant functions, while a particular PH can have more than one enzymatic activity (different substrates). For example, some PHs play an important role in rod shape maintenance and daughter cell separation or daughter cell separation and autolysis [40,49]. The precise mechanisms by which PHs are controlled are largely unknown due to the complexity of the systems involved [40,49].

Here, we found that Δ*clhAB*_*2*_ mutant cells produced abnormal short chains and wide cells in the presence of glucose during late exponential and transition growth phases (Figs 6 and 7, S3 Fig), while Δ*clhAB*_*2*_ cells showed accelerated autolysis under autolysis-inducing conditions compared to wild-type cells (Fig 8). Overall, we obtained evidence to confirm that *Bc clhAB*_*2*_ operon modulates PH activities, which are required for proper cell shape and proper chain length during cell growth and down-regulates autolysins activity.

### Chain lengthening in the presence of glucose

*B*. *cereus* chaining has been addressed in different growth conditions and environnements [37–39], but the underlying molecular mechanisms remain to be elucidated. Here, we show that *B*. *cereus clhAB*_*2*_ operon is involved in the formation of characteristic long chains in LBG growth medium at *t*_0_. Quantitative fluorescence microscopy was used to assess chain length and inter-constriction cell arrangement differences. We have first focused on total cell number present in a chain as this simple morphological measure -chain length- has been widely used in several chaining studies [50,51]. We were able to identify short chain phenotype in the absence of glucose and long chain phenotype in the presence of glucose. However, we found that the biological variation of the chain length measure was important for all analysed bacterial populations (box plot graphs, Fig 6C and S1 Fig). Thus, we searched for an additional morphological measure for chain length evaluation. As described in *B*.*anthracis* [52,53], the *Bc* chains present several constrictions that are spaced two to eight cells apart. We were able to identify two relevant inter-constriction cell-numbers: four in the absence of glucose and eight in the presence of glucose. These observations allow us to assess two inter-constriction cell arrangements, namely "short" (2–4 cells) and "long" (6–8 cells). This new measure is an indirect way to evaluate chain length and allow us to differentiate clearly short and long chain phenotypes. In addition, data acquisition was faster and easier; indeed, inter-constriction cell number counting requires fewer images because full chain images are not necessary and it was easier to generate large samples (N ≥ 200 arrangements vs. N ≥ 90 chains in our study). Moreover, we also demonstrated that chain lengthening was observed in glucose-grown cells in LB buffered with MOPS (S1 Fig). Our results show that the presence of glucose but not metabolic acid production was involved in chain lengthening in *Bc* strain. To conclude, we provide evidence that, through tight regulation by CodY and CcpA, the *clhAB*_*2*_ operon of *B*. *cereus* enables glucose-grown cells to maintain proper cell-chain lengths and cell size at the onset of the transition growth phase.

## Supporting information

S1 Fig Expression of *clhAB*_*2*_ and morphological changes of *Bacillus cereus* ATCC 14579 (*Bc*), Δ*clhAB*_*2*_ mutant (Δ), complemented mutant (ΔΩ) cell-chains in LB buffered with or without glucose. (A) Division septa and cytoplasmic membranes were imaged using the FM4-64 lipophilic dye. Top row, from the left: fluorescent micrographs of *Bc*, Δ, and ΔΩ chains at *t*_0_ in LB-MOPS medium and then in LB-MOPS medium with glucose 0.35%. Lower row: same order, phase-contrast images. Images of chains revealed strong constrictions (deeper invaginations) corresponding to cells undergoing separation. Scale bar (5μm) is shown for each image. (B) Box plots of chain length (number of cells per chain) at *t*_0_ in *Bc* (blue), Δ*clhAB*_*2*_ (red), Δ*clhAB*_*2*_Ω*clhAB*_*2*_ (green) populations. 90 chains from two independent cultures were analysed. Median (strong line in the box), interquartile range (IQR; box), whiskers (1.5 x IQR) and outliers (dot) are presented. Significance is based on two tests, Mann-Whitney and Two-Sample Fisher-Pitman Permutation, with a *P* of <0.01**. (C) Distributions of “short” (≤4) and “long” (>4) inter-constriction cell types in the *Bc*, Δ*clhAB*_*2*_, and complemented mutant populations (N = 200 cell arrangements). Two inter-constriction arrangement types in *Bc*, Δ*clhAB*_*2*_, and Δ*clhAB*_*2*_Ω*clhAB*_*2*_ populations were defined (see Materials and methods). The first type, containing cell arrangements with two to four cells ("short") and the second type, including cell arrangements with six to eight cells ("long"). (D,E) Cells of *Bc*, isogenic mutant strains (Δ*codY*, *codY*-complemented mutant, Δ*ccpA*, *ccpA-*complemented mutant) which all harbored the P_*clhAB2*_’-*lacZ* fusion, were grown in LB-MOPS medium without (closed symbols) or with 0.35% glucose (open symbols). Exponentially growing cultures of *B*. *cereus* were inoculated into standard LB medium [13] buffered with 50mM MOPS (3-(N-morpholino-propanesulfonic acid) (pH7.7± 0.2) or LB MOPS supplemented with 0.35% glucose at a final optical density of 0.05.
(TIFF)

S2 Fig CodY represses *oppA* gene *(BC2026)* expression in the presence or absence of glucose. Cells of *B*. *cereus* ATCC 14579 (*Bc*) and isogenic mutant strain Δ*codY*, which all harbored the P_*oppa (BC2026)*_’-*lacZ* fusion, were grown in LB medium without (closed symbols) or with 0.35% glucose (open symbols). Samples were harvested at the indicated times and were assayed for β-galactosidase specific activity. Glucose was added, when appropriate, at the onset of the culture. *t*_n_ is the number of hours before (-) or after *t*_0_. Representative experiment of *n = *2 experiments are shown. pHT304-P_*oppA*’_-*lacZ* (BC2026) was obtained by inserting the DNA region upstream (corresponding to the intergenic region) of the *Bc oppA* gene between the *Pst*I and *Xba*I cloning sites of pHT304-18Z. The resulting plasmid was then transferred into *B*. *cereus* by electroporation.
(TIFF)

S3 Fig Δ*clhAB*_*2*_ mutant cells produced abnormal short chains and wide cells in the presence of glucose. Phase-contrast images of *Bacillus cereus* ATCC 14579 (*Bc*) and Δ*clhAB*_*2*_ mutant (Δ) chains at *t*_-1_, *t*_0_ and *t*_2_. The onset of the transition growth phase (*t*_0_) was defined as the breakpoint in the slope of the log phase growth curve, and tn is the number of hours before (-) or after time zero [14]. One hour before the start of transition phase (*t*_-1_), and two hours after *t*_0_ (*t*_2_). LBG, LB medium with glucose 0.35%. Scale bar is 10 μM. Bacterial aliquots were removed from an exponential or early stationary phase cultures and observed with a Zeiss Axio Observer.Z1 inverted fluorescence microscope equipped with a Zeiss AxioCam MRm digital camera. Phase-contrast images were processed with Zeiss ZEN 2–lite software.
(TIFF)

S4 Fig Images of *Bacillus cereus* ATCC 14579 and Δ*clhAB*_*2*_ mutant chains and visualization of peritrichous flagella. Flagella, septa and constrictions were visualized using transmission electronic microscopy (TEM) after negative staining of bacteria. The sequential two-droplet method was used. For each condition, 1 ml of early post-exponential cells (OD between 3 and 4) grown in LB medium with glucose 0.35% was washed 2 times by centrifugation and resuspended and concentrated in 100 μl with PBS 1X. Mesh formvar carbon coated nickel grids (Electron Microscopy Sciences, LFG distribution, France) were used and bacteria bind to grid by adsorption. Then, for staining, a 1% (w/v) phosphotungstic acid (Sigma-Aldrich, USA) was used. Observations were performed using an HT7700 transmission electron microscope (Hitachi, Japan) equipped with an 8 million pixels format CCD camera driven by the image capture engine software AMT, version 6.02, at the INRA MIMA2 microscopy platform (Jouy-en-Josas, France). Images were made at 80 kV in high contrast mode with an objective aperture adjusted for each sample and magnification.
(TIFF)

S5 Fig Expression of *clhAB*_*2*_ in the presence of three different sugars and in the presence of various concentrations of glucose. Cells of *Bc* which harbored the transcriptional P_*clhAB2*_’-*lacZ* fusion construct, were grown in LB (closed symbols) or in LB with sugar (open symbols) media. Samples were harvested at the indicated times and were assayed for β-galactosidase specific activity. (A) Fructose, glucose or sucrose and (B) different glucose concentrations (0.3%-0.6% 1%) were added at the onset of the culture. Time zero corresponds to the entry into the transition growth phase. The data presented are representative of three independent experiments.
(TIFF)
