# Supplementary figures and images for: The CodY-dependent clhAB2 operon is involved in cell shape, chaining and autolysis in Bacillus cereus ATCC 14579
Source: PLoS One. 2017 Oct 9;12(10):e0184975. doi: 10.1371/journal.pone.0184975 (PMC5633148; doi:10.1371/journal.pone.0184975)

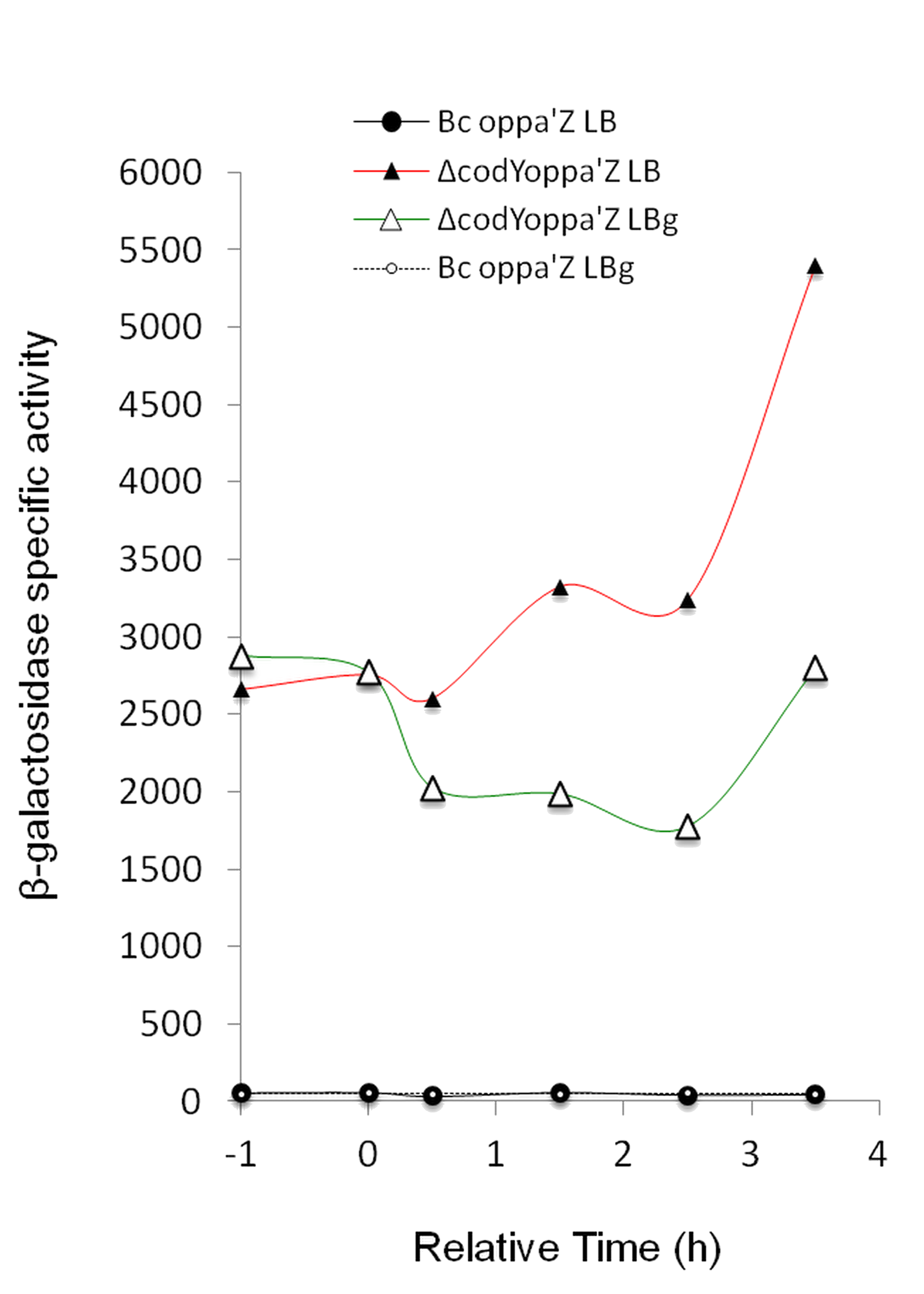

Supplement: S1 Fig — (A) Division septa and cytoplasmic membranes were imaged using the FM4-64 lipophilic dye. Top row, from the left: fluorescent micrographs of Bc, Δ, and ΔΩ chains at t0 in LB-MOPS medium and then in LB-MOPS medium with glucose 0.35%. Lower row: same order, phase-contrast images. Images of chains revealed strong constrictions (deeper invaginations) corresponding to cells undergoing separation. Scale bar (5μm) is shown for each image. (B) Box plots of chain length (number of cells per chain) at t0 in Bc (blue), ΔclhAB2 (red), ΔclhAB2ΩclhAB2 (green) populations. 90 chains from two independent cultures were analysed. Median (strong line in the box), interquartile range (IQR; box), whiskers (1.5 x IQR) and outliers (dot) are presented. Significance is based on two tests, Mann-Whitney and Two-Sample Fisher-Pitman Permutation, with a P of <0.01**. (C) Distributions of “short” (≤4) and “long” (>4) inter-constriction cell types in the Bc, ΔclhAB2, and complemented mutant populations (N = 200 cell arrangements). Two inter-constriction arrangement types in Bc, ΔclhAB2, and ΔclhAB2ΩclhAB2 populations were defined (see Materials and methods). The first type, containing cell arrangements with two to four cells ("short") and the second type, including cell arrangements with six to eight cells ("long"). (D,E) Cells of Bc, isogenic mutant strains (ΔcodY, codY-complemented mutant, ΔccpA, ccpA-complemented mutant) which all harbored the PclhAB2’-lacZ fusion, were grown in LB-MOPS medium without (closed symbols) or with 0.35% glucose (open symbols). Exponentially growing cultures of B. cereus were inoculated into standard LB medium [13] buffered with 50mM MOPS (3-(N-morpholino-propanesulfonic acid) (pH7.7± 0.2) or LB MOPS supplemented with 0.35% glucose at a final optical density of 0.05. (TIFF) [file pone.0184975.s001.tiff]

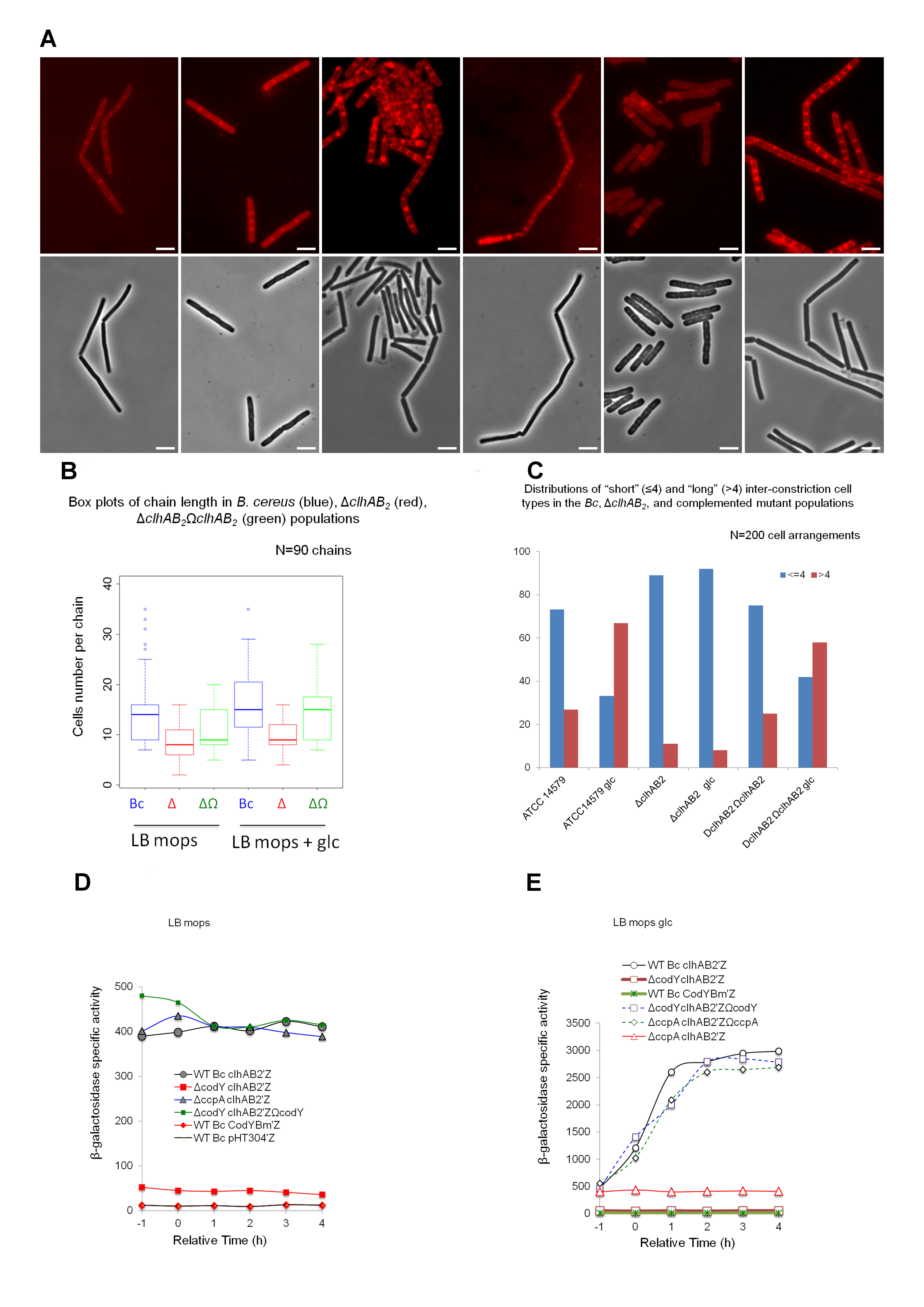

Supplement: S2 Fig — Cells of B. cereus ATCC 14579 (Bc) and isogenic mutant strain ΔcodY, which all harbored the Poppa (BC2026)’-lacZ fusion, were grown in LB medium without (closed symbols) or with 0.35% glucose (open symbols). Samples were harvested at the indicated times and were assayed for β-galactosidase specific activity. Glucose was added, when appropriate, at the onset of the culture. tn is the number of hours before (-) or after t0. Representative experiment of n = 2 experiments are shown. pHT304-PoppA’-lacZ (BC2026) was obtained by inserting the DNA region upstream (corresponding to the intergenic region) of the Bc oppA gene between the PstI and XbaI cloning sites of pHT304-18Z. The resulting plasmid was then transferred into B. cereus by electroporation. (TIFF) [file pone.0184975.s002.tiff]

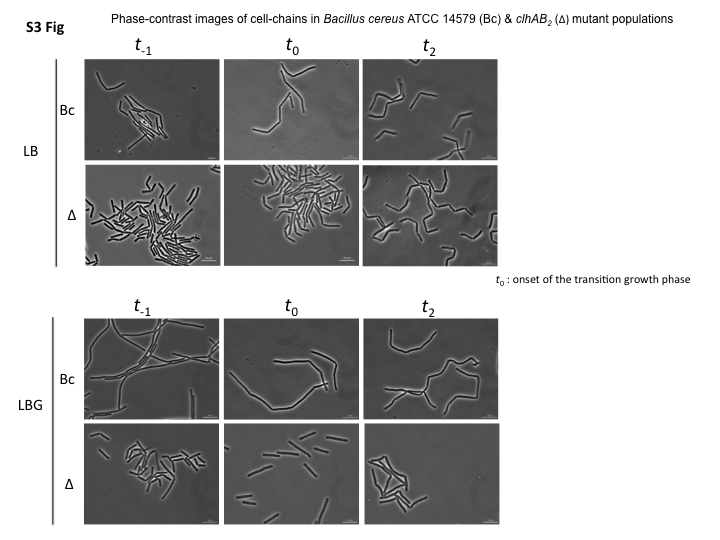

Supplement: S3 Fig — Phase-contrast images of Bacillus cereus ATCC 14579 (Bc) and ΔclhAB2 mutant (Δ) chains at t-1, t0 and t2. The onset of the transition growth phase (t0) was defined as the breakpoint in the slope of the log phase growth curve, and tn is the number of hours before (-) or after time zero [14]. One hour before the start of transition phase (t-1), and two hours after t0 (t2). LBG, LB medium with glucose 0.35%. Scale bar is 10 μM. Bacterial aliquots were removed from an exponential or early stationary phase cultures and observed with a Zeiss Axio Observer.Z1 inverted fluorescence microscope equipped with a Zeiss AxioCam MRm digital camera. Phase-contrast images were processed with Zeiss ZEN 2–lite software. (TIFF) [file pone.0184975.s003.tiff]

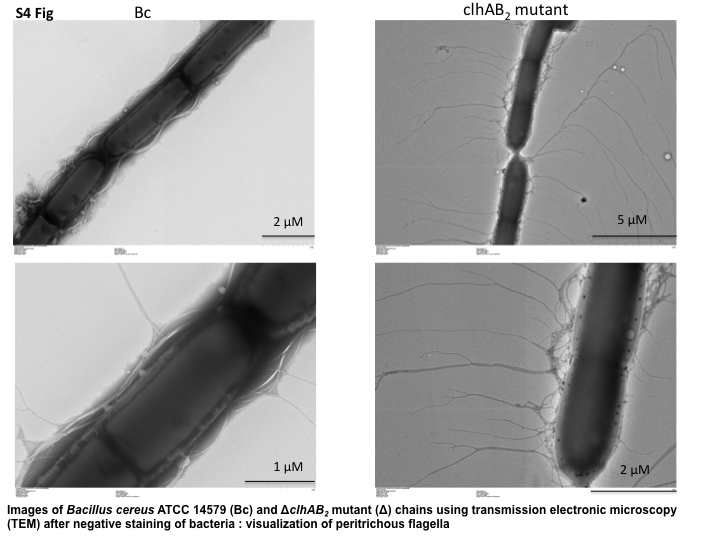

Supplement: S4 Fig — Flagella, septa and constrictions were visualized using transmission electronic microscopy (TEM) after negative staining of bacteria. The sequential two-droplet method was used. For each condition, 1 ml of early post-exponential cells (OD between 3 and 4) grown in LB medium with glucose 0.35% was washed 2 times by centrifugation and resuspended and concentrated in 100 μl with PBS 1X. Mesh formvar carbon coated nickel grids (Electron Microscopy Sciences, LFG distribution, France) were used and bacteria bind to grid by adsorption. Then, for staining, a 1% (w/v) phosphotungstic acid (Sigma-Aldrich, USA) was used. Observations were performed using an HT7700 transmission electron microscope (Hitachi, Japan) equipped with an 8 million pixels format CCD camera driven by the image capture engine software AMT, version 6.02, at the INRA MIMA2 microscopy platform (Jouy-en-Josas, France). Images were made at 80 kV in high contrast mode with an objective aperture adjusted for each sample and magnification. (TIFF) [file pone.0184975.s004.tiff]

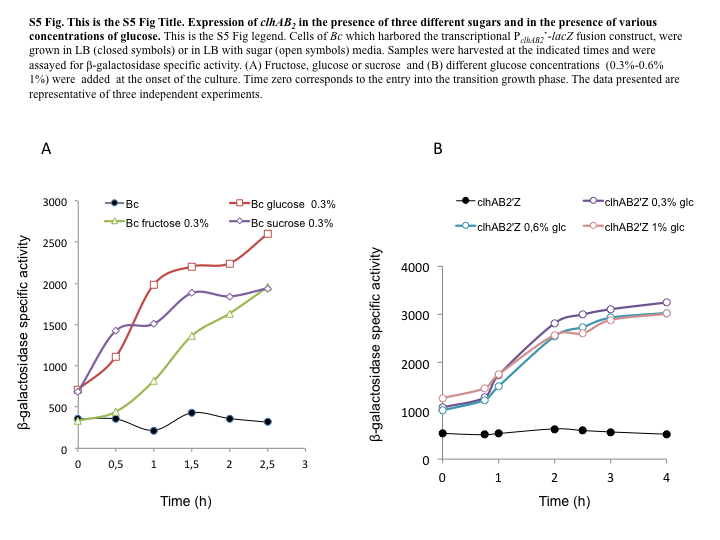

Supplement: S5 Fig — Cells of Bc which harbored the transcriptional PclhAB2’-lacZ fusion construct, were grown in LB (closed symbols) or in LB with sugar (open symbols) media. Samples were harvested at the indicated times and were assayed for β-galactosidase specific activity. (A) Fructose, glucose or sucrose and (B) different glucose concentrations (0.3%-0.6% 1%) were added at the onset of the culture. Time zero corresponds to the entry into the transition growth phase. The data presented are representative of three independent experiments. (TIFF) [file pone.0184975.s005.tiff]
